# Applications and prospects of biomaterials in diabetes management

**DOI:** 10.3389/fbioe.2025.1547343

**Published:** 2025-03-07

**Authors:** Wenhe Guan, Liang Zhang

**Affiliations:** ^1^ Department of Pediatrics, Shengjing Hospital of China Medical University, Shenyang, Liaoning, China; ^2^ Department of Human Anatomy, School of Basic Medicine, Shenyang Medical College, Shenyang, Liaoning, China

**Keywords:** biomaterials, diabetes, insulin delivery, tissue engineering, biosensors

## Abstract

Diabetes is a widespread metabolic disorder that presents considerable challenges in its management. Recent advancements in biomaterial research have shed light on innovative approaches for the treatment of diabetes. This review examines the role of biomaterials in diabetes diagnosis and treatment, as well as their application in managing diabetic wounds. By evaluating recent research developments alongside future obstacles, the review highlights the promising potential of biomaterials in diabetes care, underscoring their importance in enhancing patient outcomes and refining treatment methodologies.

## 1 Introduction

Diabetes mellitus is a chronic disease marked by prolonged hyperglycemia, which arises from defects in insulin secretion, insulin action, or a combination of both (American Diabetes Association, 2014; Chaudhury et al., 2017; Defronzo, 2009). The primary types of diabetes include Type 1 diabetes mellitus (T1DM) and Type 2 diabetes mellitus (T2DM). T1DM is mainly an autoimmune condition leading to the destruction of insulin-producing beta cells in the pancreas (Khaiz et al., 2025; Nyaga et al., 2018a; Nyaga et al., 2018b). In contrast, T2DM is often linked to insulin resistance, influenced by lifestyle factors and genetic predispositions (Dariya et al., 2019; Ghasemi and Norouzirad, 2019; Memon et al., 2022). The incidence of diabetes worldwide has been on a steady rise, resulting in significant public health implications, particularly as demographic trends lean towards aging populations and lifestyle changes, including increased obesity rates (Cano-Ibanez and Bueno-Cavanillas, 2024). The International Diabetes Federation reported that approximately 537 million adults were diagnosed with diabetes in 2021, with projections indicating a rise to 783 million individuals by 2045 (Klangjareonchai et al., 2021).

Conventional diabetes management approaches include pharmacological treatments such as insulin and oral hypoglycemic agents, as well as lifestyle changes encompassing diet and exercise (Deng et al., 2018). Nonetheless, achieving optimal glycemic control remains a challenge for numerous patients, often due to factors like medication adherence, the complexity of treatment protocols, and the psychosocial burdens associated with the disease (Al-Qerem et al., 2022; Summers-Gibson, 2021). These challenges highlight the urgent need for innovative strategies in diabetes management (Kalra et al., 2022).

In recent times, the application of biomaterials has surfaced as a promising pathway for the enhancement of diabetes treatment and management (Aldahish et al., 2024; Emad et al., 2024; Nemati et al., 2023). This review seeks to investigate the diverse applications of biomaterials within the realm of diabetes management, addressing their potential to mitigate the limitations of existing treatment methodologies while improving the quality of life for individuals with diabetes. The evolving role of biomaterials in diabetes management marks a significant advancement in addressing the complexities inherent to this chronic condition (Iqbal et al., 2023).

## 2 Diagnosis of diabetes using biomaterial-mediated strategies

Biosensors have become essential instruments across various domains, particularly in healthcare. Within this sector, they provide rapid and precise monitoring of biological parameters (Kim et al., 2019; Li et al., 2023; Yoon et al., 2020). These sensors possess the capability to detect specific biological markers molecules, delivering crucial real-time information essential for the diagnosis, management, and prevention of diseases (Kong et al., 2024; Xing et al., 2024).

Conventional diagnostic approaches for diabetes, which largely rely on fasting plasma glucose (FPG), oral glucose tolerance tests (OGTT), and hemoglobin A1c (HbA1c) assessments, exhibit several shortcomings. These techniques are susceptible to various influences, such as stress, illness, and inconsistencies in laboratory procedures, which may result in misdiagnosis or delays in diagnosis (Young et al., 2023). For instance, HbA1c levels may not provide an accurate representation of glycemic control in specific populations, including those with hemoglobinopathies or individuals who have recently received blood transfusions (Bhatti et al., 2024). Traditional glucose testing methods, primarily based on blood glucose meters, encounter numerous challenges that hinder patient adherence and effective diabetes management. Ahmadian et al. conducted a comprehensive review of current technologies, comparing the benefits and drawbacks of both invasive and non-invasive glucose monitoring techniques (Ahmadian et al., 2023). Many of these methods necessitate finger-pricking, which can be painful and inconvenient, resulting in many patients opting to forgo regular testing (Burge, 2001). Furthermore, the precision of blood glucose meters can be influenced by several factors, including user error, calibration discrepancies, and environmental conditions, leading to variable readings (Tankasala and Linnes, 2019). Additionally, conventional testing methods typically offer only a snapshot of glucose levels at a single moment, failing to account for fluctuations that occur throughout the day. The psychological strain associated with diabetes management, including the stress from frequent monitoring and apprehension regarding complications, highlights the demand for reliable and minimally invasive glucose testing methods (Xie et al., 2023).

The significance of glucose monitoring sensors in diabetes management cannot be overstated. Recent advancements in biosensor technology have facilitated the development of non-invasive and continuous glucose monitoring systems that enhance patient adherence and improve health outcomes (Dua et al., 2024; Hina and Saadeh, 2020; Teymourian et al., 2020). The integration of biosensors with mobile technology and data analytics platforms has further increased their utility, allowing for continuous monitoring and remote health management (Arun et al., 2024; Bent et al., 2020).

### 2.1 Detection by sensors composed of nanomaterials in conjunction with Raman spectroscopy

Biomedical nanomaterials, particularly those engineered for glucose sensing, have demonstrated promising advancements in improving the sensitivity and specificity of diabetes diagnostics. For example, electrospun nanofibers have emerged as a novel category of functional nanocomposites exhibiting remarkable biosensing capabilities (Du et al., 2022). The incorporation of nanomaterials, such as gold nanoparticles and carbon nanotubes, has further enhanced the efficacy of biosensors, enabled the simultaneous detection of multiple analytes and accelerated response times (Nisar et al., 2024; Otero and Magner, 2020; Putzbach and Ronkainen, 2013). Moreover, when combined with organometallic compounds, these nanomaterials can significantly augment the performance of Raman spectroscopy, allowing for the detection of subtle spectral variations related to diabetes biomarkers. This synergistic approach not only improves detection sensitivity but also extends the range of potential applications in clinical diagnostics (Jagannathan et al., 2023).

The high surface area of these nanomaterials promotes increased loading of recognition elements, leading to enhanced detection capabilities. Enhanced performance of biosensors has been documented (Mousavi et al., 2022). Furthermore, nanomaterials can be tailored to respond to specific stimuli, facilitating the creation of intelligent biosensors capable of real-time monitoring of physiological variations (Scandurra et al., 2023). Recent innovations utilizing DNA nanostructures have demonstrated significant potential in biosensing applications, where they can be engineered for the selective binding of target molecules, thus improving detection specificity (Mohammad, 2024). Ongoing investigations in this field continue to reveal novel opportunities for the application of nanomaterials in biosensing, which may lead to the development of groundbreaking diagnostic tools for clinical use.

Raman spectroscopy operates on the principle of inelastic scattering of monochromatic light, typically emitted by a laser. When light interacts with the vibrations of molecules, it can scatter with a shift in energy that corresponds to the vibrational modes of those molecules. This characteristic renders Raman spectroscopy a versatile instrument for both qualitative and quantitative analyses across various applications, including the identification of biomarkers for diseases such as diabetes (Xie et al., 2023). A prominent example of this technique’s efficacy is its application in measuring urinary albumin levels, a critical biomarker for diabetic kidney disease. Research has illustrated that Raman spectroscopy can effectively identify specific spectral peaks linked to albumin concentrations in urine samples from individuals diagnosed with type 2 diabetes, indicating its potential for non-invasive monitoring of renal complications related to diabetes (Flores-Guerrero et al., 2020). Moreover, Raman spectroscopy has been employed to investigate retinal tissue for early indicators of diabetic retinopathy, offering insights into the biochemical alterations occurring in the retina due to prolonged hyperglycemia. The capacity of this technique to distinguish between healthy and diseased tissues through spectral analysis renders it an invaluable tool for early diagnosis and timely intervention in diabetic patients (Chen et al., 2021). Furthermore, advancements in machine learning algorithms applied to Raman spectral data have bolstered the precision of diabetes detection, highlighting the technology’s potential to transform diabetes management and enhance patient outcomes (Chen et al., 2024e).

**FIGURE 1:**
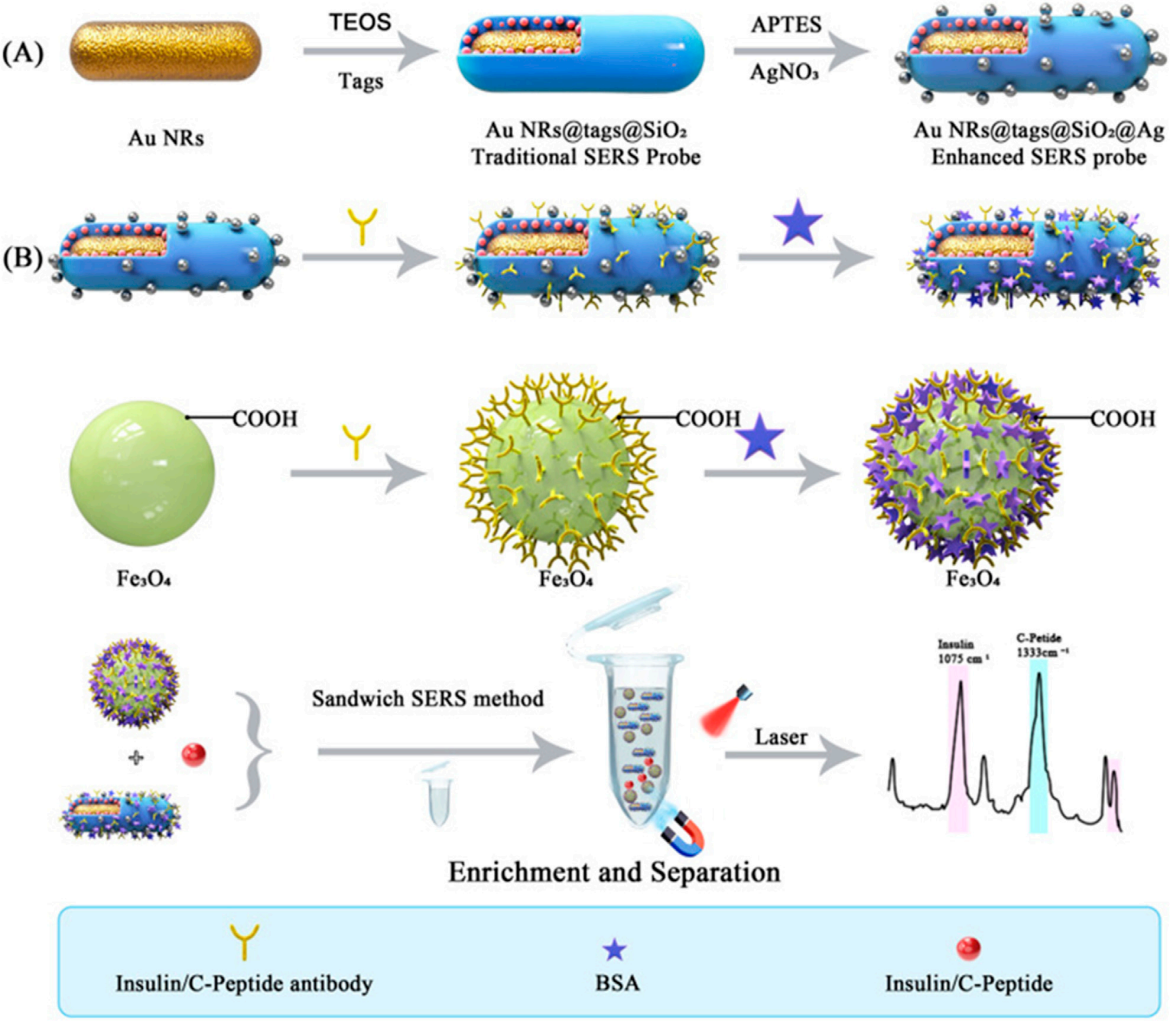
Depicts the schematic representation of **(A)** the synthesis of the SERS probe and **(B)** the SERS-based immunoassay utilized for the detection of insulin antibodies and C-peptide antibodies. Reproduced with permission from Zhang et al. (2024b).

The amalgamation of biomedical nanomaterials with Raman spectroscopy presents numerous advantages while also posing significant challenges (Oliveira et al., 2022). A primary advantage of this integration lies in the enhancement of diagnostic accuracy and sensitivity. For instance, a core-shell structure of Au nanorods@Raman tags@SiO2@Ag nanocomposite has been synthesized and employed for the surface-enhanced Raman scattering (SERS) detection of insulin and C-peptide in trace serum (Zhang et al., 2024b). This is illustrated in Figure 1.

### 2.2 Detection via gas sensors composed of biomedical metal oxides

Exhalation detection technology has attracted notable interest due to its non-invasive and convenient nature, particularly in the regulation of glucose levels, which is essential for managing conditions such as diabetes. In breath analysis, glucose is often detected indirectly through its metabolic byproducts, including acetone, which is produced during the metabolism of fatty acids when glucose levels are diminished (Galassetti et al., 2005; Hwang et al., 2021; Li et al., 2015). The well-established relationship between breath acetone and blood glucose levels provides a foundation for the development of sensors capable of measuring glucose levels via breath analysis (Kalidoss and Umapathy, 2019; Righettoni et al., 2013; Tanda et al., 2014). The accurate and prompt identification of acetone is vital for maintaining safety in industrial production and for the clinical assessment of diabetes. Consequently, the advancement of high-performance acetone sensors has become increasingly significant (Guan et al., 2025) (Table 1). Analyzing breath can facilitate real-time observation of metabolic alterations, enabling timely interventions to avert conditions such as hyperglycemia or hypoglycemia (Xie et al., 2023). Moreover, breath testing is characterized by its convenience and discretion, which enhances patient adherence and promotes ongoing monitoring during daily activities (Vajhadin et al., 2021).

**TABLE 1:** Overview of gas sensors utilizing biomedical metal-oxides for acetone detection in 2024.

Composition of biomaterials	Advantage	Application	References
α-Fe_2_O_3_-multiwalled carbon nanotube (MWCNT) nanocomposite	detect acetone in exhaled breath under high humidity	diabetes detection	Ansari et al. (2024)
a K/Sn-Co_3_O_4_ porous microsphere	without the removing water vapor from exhaled breath	diabetes detection	Na et al. (2024)
porous 2D WO_3_ nanosheets	1. The rapid diffusion and adsorption of acetone molecules 2. Higher charge activity and adsorption capacity	diabetes detection	Guan et al. (2025)
biofluorometric acetone gas sensor (bio-sniffer) using secondary alcohol dehydrogenase	Sub-ppbv Level Sensitivity (quantitative characteristics in the concentration range of 0.5–1,000 ppbv)	continuous measurement of acetone gas released through the skin	Iitani et al. (2024)
16 wt% N-CQDs/WO_3_ sensor	1. Real-time acetone detection 2. Portable human-exhaled gas sensors	diabetes detection	Ni et al. (2024)
Al/CuO:Ni nanostructured thin films	enhances the sensitivity and selectivity of acetone sensors for practical applications as breath detectors in biomedical diagnostics	1. Diabetes detection 2. Ensuring industrial safety by preventing adverse health and environmental impacts of acetone exposure	Litra et al. (2024)
bimetallic PtAu-decorated SnO_2_ nanospheres (PtAu/SnO_2_)	1. Superior sensitivity to acetone of 0.166–100 ppm at 300°C 2. Remarkable gas response, rapid response and recovery times, strong linear correlation, excellent repeatability, long-term stability, and satisfactory selectivity at 300°C	the early diagnosis and screening of diabetes	Zhu et al. (2024)
ZIF-67-derived oxide cage/nanofiber Co_3_O_4_/In_2_O_3_ heterostructure	1. Abundant reversible active adsorption/reaction sites alongside a type-I heterojunction 2.ultrasensitive response to acetone concentrations ranging from 954 to 50 ppm at 300°C 3. A low detection limit of 18.8 ppb, a swift response time of just 4 s, commendable selectivity and repeatability, acceptable resistance to humidity interference, and sustained long-term stability	environmental monitoring and the early diagnosis of diabetes	Wu et al. (2024)
bimetallic Au@Pt core-shell nanospheres (BNSs) functionalized-electrospun ZnFe_2_O_4_ nanofibers (ZFO NFs)	Compared to pure NFs-650 analogue, the ZFO NFs/BNSs-2 sensor exhibits a stronger mean response (3.32 vs. 1.84), quicker response/recovery speeds (33 s/28 s vs. 54 s/42 s), and lower operating temperature (188°C vs. 273°C) toward 0.5 ppm acetone	potential for diabetes diagnosis in individual healthcare settings	Zhao et al. (2024a)
Gd_2_Zr_2_O_7_ solid electrolyte and CoSb_2_O_6_ sensing electrode	1. A low detection limit of 10 ppb, enabling linear detection for acetone in an extremely wide range of 10 ppb–100 ppm 2. Excellent selectivity, repeatability, and stability	diagnosis and monitoring of diabetic ketosis	Jiang et al. (2024)
Porous Co(3)O(4) nanofoam	1. A low detection limit of 0.05 ppm and a high sensitivity of −56 mV/decade in the acetone concentration range of 1–20 ppm 2. Outstanding repeatability, acceptable selectivity, effective resistance to O2 and humidity, and long-term stability during continuous measurements over a duration exceeding 30 days	distinguish between healthy individuals and patients with diabetic ketosis	Hao et al. (2024b)

However, environmental factors substantially influence the detection of glucose and its metabolites in exhaled breath, impacting both the collection and analytical processes. Elements such as humidity and the presence of competing volatile compounds can hinder gas sensor performance (Esteves et al., 2022; Xie et al., 2018). Therefore, it is essential to optimize the operating conditions of sensors to mitigate these environmental effects. Zhou et al. developed a self-designed condensation device for exhaled breath, which allowed for the condensation and collection of human exhaled breath, enabling the analysis of glucose in the collected condensate via ion chromatography using a pulsed amperometric instrument (Zhou et al., 2022). For instance, custom-built exhaled breath collection devices that regulate temperature and humidity have demonstrated potential for enhancing the reproducibility of glucose measurements in breath samples (Desai et al., 2025). A noninvasive blood glucose detection apparatus that utilizes acetone sensing in exhaled breath employs an α-Fe2O3-multiwalled carbon nanotube (MWCNT) nanocomposite to accurately measure acetone levels, even in high humidity conditions (Ansari et al., 2024). Furthermore, the incorporation of nanostructured materials and composite sensors has been shown to improve sensitivity and selectivity, enabling more precise glucose detection in the presence of interfering substances found in exhaled breath (K et al., 2025). Notably, an ultrasensitive acetone gas sensor based on a K/Sn-Co3O4 porous microsphere can accurately differentiate between diabetic patients and healthy individuals based on variations in acetone concentrations without the need to eliminate water vapor from exhaled breath, highlighting its substantial potential for diabetes diagnosis (Na et al., 2024).

### 2.3 Detected by wearable sensors made of biomaterials

**FIGURE 2:**
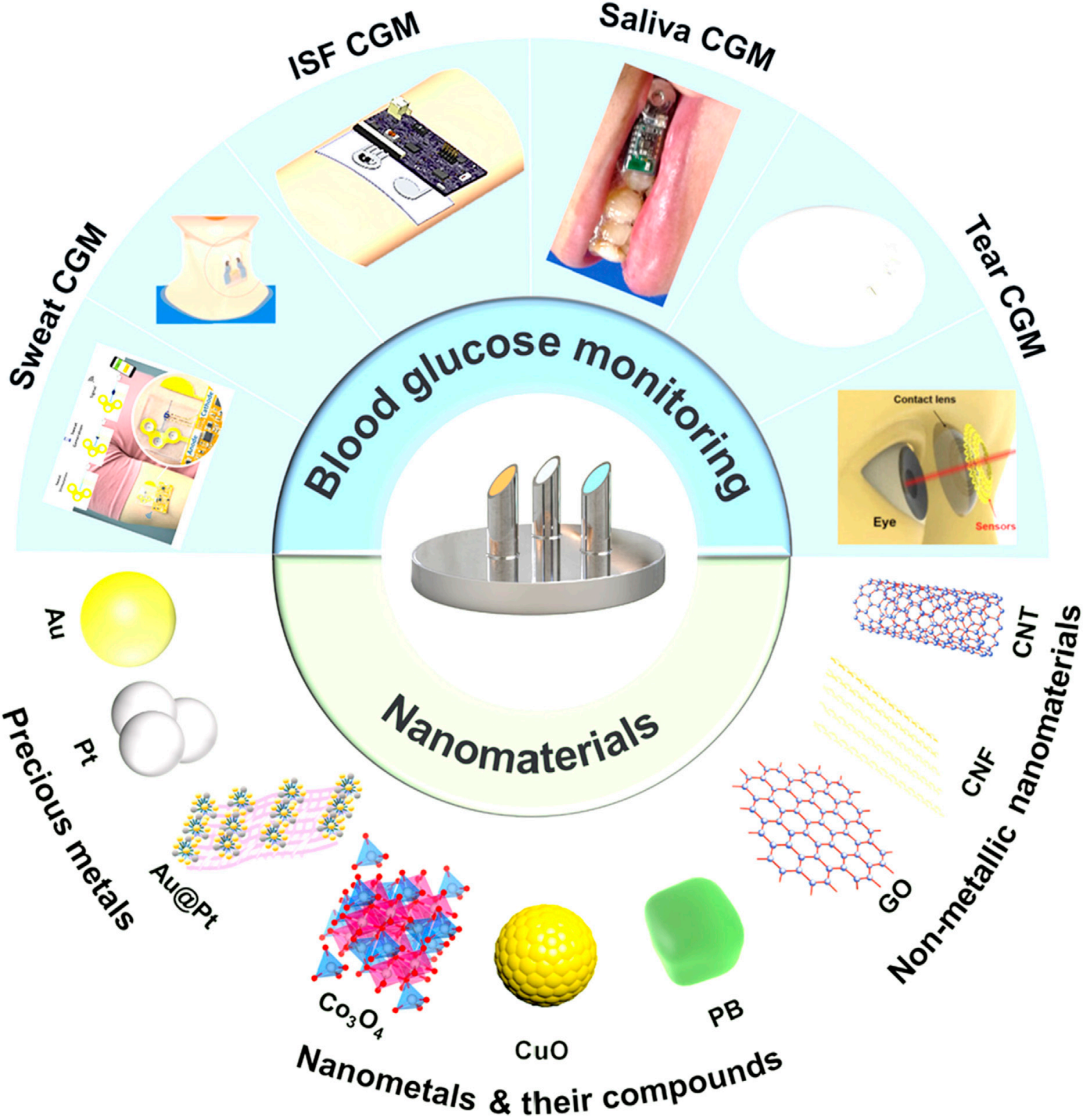
Illustrates the continuous glucose monitoring (CGM) sensors employed for the assessment of various biological fluids and the nanomaterials developed for tear glucose analysis in recent years, reproduced with permission from Zhou et al. (2024b).

**FIGURE 3:**
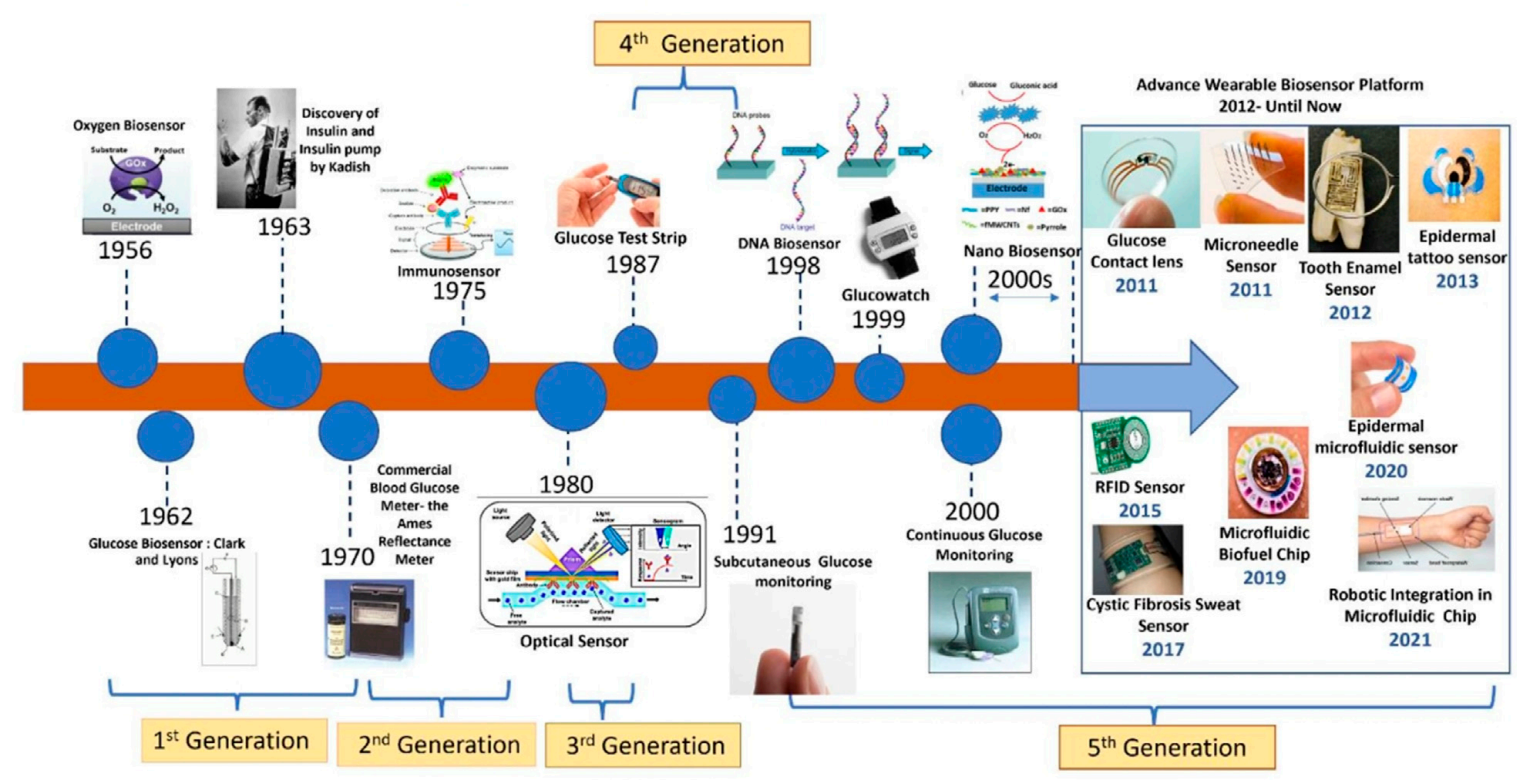
Depicts the historical progression of biosensor development for wearable technology up to 2021, reproduced with permission from Zafar et al. (2022).

Wearable sensors made from biomaterials designed for sweat glucose detection have garnered significant interest due to their capacity for continuous monitoring without the discomfort of finger-prick tests. Zhou et al. conducted a thorough review of the principles and advancements in electrochemical glucose sensors, compiling findings on various innovative nanomaterials suitable for continuous glucose monitoring (CGM) (Zhou et al., 2024b). The work illustrated the applications and construction strategies of diverse nanomaterials, including precious metals, nanometals, their compounds, and nonmetallic nanomaterials. Figure 2 in their study encapsulates these insights on CGM technology, while Figure 3 traces the evolution of biosensor development for wearables up to 2021.

These sensors utilize cutting-edge materials and designs to enhance sensitivity and selectivity, thereby enabling precise real-time glucose detection (Dervisevic et al., 2022; Zafar et al., 2022; Zhou et al., 2023). The hyaluronate (HA)-modified Au@Pt bimetallic electrodes have been validated through animal trials for their capacity to provide long-term, accurate, and robust CGMs in smart contact lenses, paving the way for continuous blood glucose monitoring (Han et al., 2023). In 2023, Zhang et al. summarized the metallic nanomaterials employed in wearable non-invasive glucose sensors, encompassing zero-dimensional (0D), one-dimensional (1D), and two-dimensional (2D) monometallic nanomaterials, as well as bimetallic configurations (Zhang et al., 2023b). In addition, Govindaraj et al. provided a thorough summary of various categories of non-enzymatic glucose sensor materials, which encompass composites, non-precious transition metals along with their respective metal oxides and hydroxides, precious metals and their alloys, carbon-based materials, conducting polymers, metal-organic framework (MOF)-based electrocatalysts, as well as glucose sensors designed for wearable devices (Govindaraj et al., 2023). Furthermore, enzyme-free nanoparticle-based glucose sensors signify a noteworthy advancement, presenting a more straightforward and cost-efficient alternative for glucose monitoring (Boucheta et al., 2024). Additionally, microfluidic devices have been engineered to assess the performance of these sensors, thereby ensuring their reliability in clinical environments (Yunos et al., 2021). Zhang et al. introduced a handheld biosensor capable of detecting acetone through fluorescence, utilizing the enzymatic reaction of secondary alcohol dehydrogenase (S-ADH) in conjunction with β-nicotinamide adenine dinucleotide (NADH, λex = 340 nm, λem = 490 nm). This device, characterized by its portability and high sensitivity and selectivity, is anticipated to see extensive application in clinical diagnostics as well as in the realm of wearable biochemical sensors in the forthcoming years (Zhang G. et al., 2025). As advancements in these technologies continue, they hold the potential to revolutionize diabetes management, enabling patients to achieve optimal glucose levels with enhanced convenience.

## 3 Treatment of diabetes through biomaterial-mediated strategies

Insulin plays a pivotal role in the management of diabetes, necessitating effective delivery mechanisms. The utilization of biomaterials, known for their exceptional biocompatibility, degradability, and distinctive functional properties, is essential in this context. Such materials significantly enhance insulin stability, modulate its release kinetics, and facilitate targeted delivery, thereby offering a safer and more efficient therapeutic option for individuals with diabetes. Novel biomaterial carriers can transport antidiabetic drugs to address different types of diabetes (Table 2).

**TABLE 2:** The advantages of various insulin drug delivery vectors.

Vector		Preparation method	Advantage	Treatment object	Treatment stage	References
Microneedle		3D printing and mold-based methods	penetrates the outer layer of the skin without reaching the nerve endings, facilitating sustained insulin release	T1DM, T2DM, and in some cases, used when traditional injection methods cause excessive pain or skin damage in diabetic patients	Initial treatment stage to establish stable insulin delivery, or when patients have difficulties with traditional injection methods	Jin et al. (2018), Xu et al. (2024), Zhao et al. (2024b) Razzaghi et al. (2024) Chen et al. (2019), Lin et al. (2025a), Lin et al. (2025b), Wang et al. (2020)
ASMNs@PVP-INS		antimicrobial sponge MNs (ASMNs@PVP-INS) modified with polyvinylpyrrolidone (PVP)	excellent mechanical strength, effectively maintaining glucose control without inducing hypoglycemia, no significant toxicity to mice	T1DM, T2DM	Not mentioned	Zhang et al. (2025b)
insulin transmitter		ultrasound, microjet method	encapsulation rate >80%, good stability, strong deformation and good transdermal performance	T1DM, T2DM, especially suitable for patients who are reluctant to use injection methods and have relatively good skin conditions	Any stage of diabetes treatment where non-invasive insulin delivery is preferred	Jarosinski et al. (2021)
biodegradable polymer material	Chitosan and its derivatives	emulsification-chemical crosslinking method, spray drying, solvent volatilization method, etc	good biocompatibility, degradability, film-forming or spheroidal properties	T1DM, T2DM, and can be used in diabetic wound healing scenarios	Drug delivery stage in diabetes treatment, and applied in wound care for diabetic patients	Abozaid et al. (2023), Lupascu et al. (2024), Mohamed et al. (2021), Morcol et al. (2004) Ali and Lehmussaari (2006) Baek et al. (2012) Blagden et al. (2007) Aldahish et al. (2024), Ali et al. (2009)
	silk fibroin			T1DM, T2DM, may have potential in long-term insulin storage and delivery due to its unique properties	Potentially used in the stage of developing long - acting insulin formulations	
	polylactose			T1DM, T2DM, might be suitable for patients with specific metabolic requirements	May be involved in the formulation of certain sustained - release insulin products	
	Hydroxyapatite			T1DM, T2DM, can be used in combination with insulin for bone - related diabetes complications	Treatment stage for diabetic patients with bone - related problems	
INS-NPs		ionotropic pre-gelation followed by polyelectrolyte complexation technique	Strengthen drug stability and improve bioavailability; achieve targeted drug release to reduce the toxic and side effects of drugs on the body; control the amount of drug release to make the effect of drugs in the body more obvious	T1DM, T2DM, especially useful when precise control of insulin release is needed	Advanced treatment stage where more refined insulin delivery is required	Kassem et al. (2017)
Chitosan/cyclodextrin nanoparticles		Ionic gel technology	Stable for at least 4 h at simulated intestinal fluid pH 6.8 and 37° C	T1DM, T2DM, beneficial for oral insulin delivery attempts	Exploratory stage of developing oral insulin delivery systems	Krauland and Alonso (2007)
Methocel-lipid hybrid nanocarriers		Methocel was added to solid lipid nanoparticles (SLN) to form	Good biocompatibility, low cytotoxicity, good drug protection, and good interaction with cells, while overcoming its key limitations in effectively encapsulating peptides	T1DM, T2DM, applicable when enhancing the interaction between insulin and cells is necessary	Treatment stage focused on improving the efficacy of insulin at the cellular level	Boushra et al. (2016)
6-O-vinyl sebacic acid-D-galactopyranosyl ester block 3-acrylamide phenylboric acid p (OVNG-b-AAPBA)		block copolymer	With optimal molecular weight and thermal stability, the prepared nanoparticles can be used in drug delivery systems. The prepared nanoparticles have good morphology and their safety has been verified by MTT and chronic animal toxicology tests. The drug loading rate and encapsulation efficiency increase with the increase of AAPBA content in the polymer, which can effectively maintain blood sugar in diabetic mice for 96 h	T1DM, T2DM, effective in maintaining stable blood sugar levels over an extended period	Treatment stage aiming for long - term blood sugar control	Zhong et al. (2020)
chitosan nanoparticle/poly (vinyl alcohol) (PVA) hybrid HGs (CPHGs)		PVA and chitosan nanoparticles (CNPs) are cross-linked with a glucose-responsive formylphenylboronic acid (FPBA)-based cross-linker *in situ*	in vitro drug release assay reveals size-dependent glucose-responsive drug release from the CPHGs under physiological conditions	T1DM	T1DM rat model and in vitro	Ali et al. (2023)

### 3.1 Delivery via nanoparticles

**FIGURE 4:**
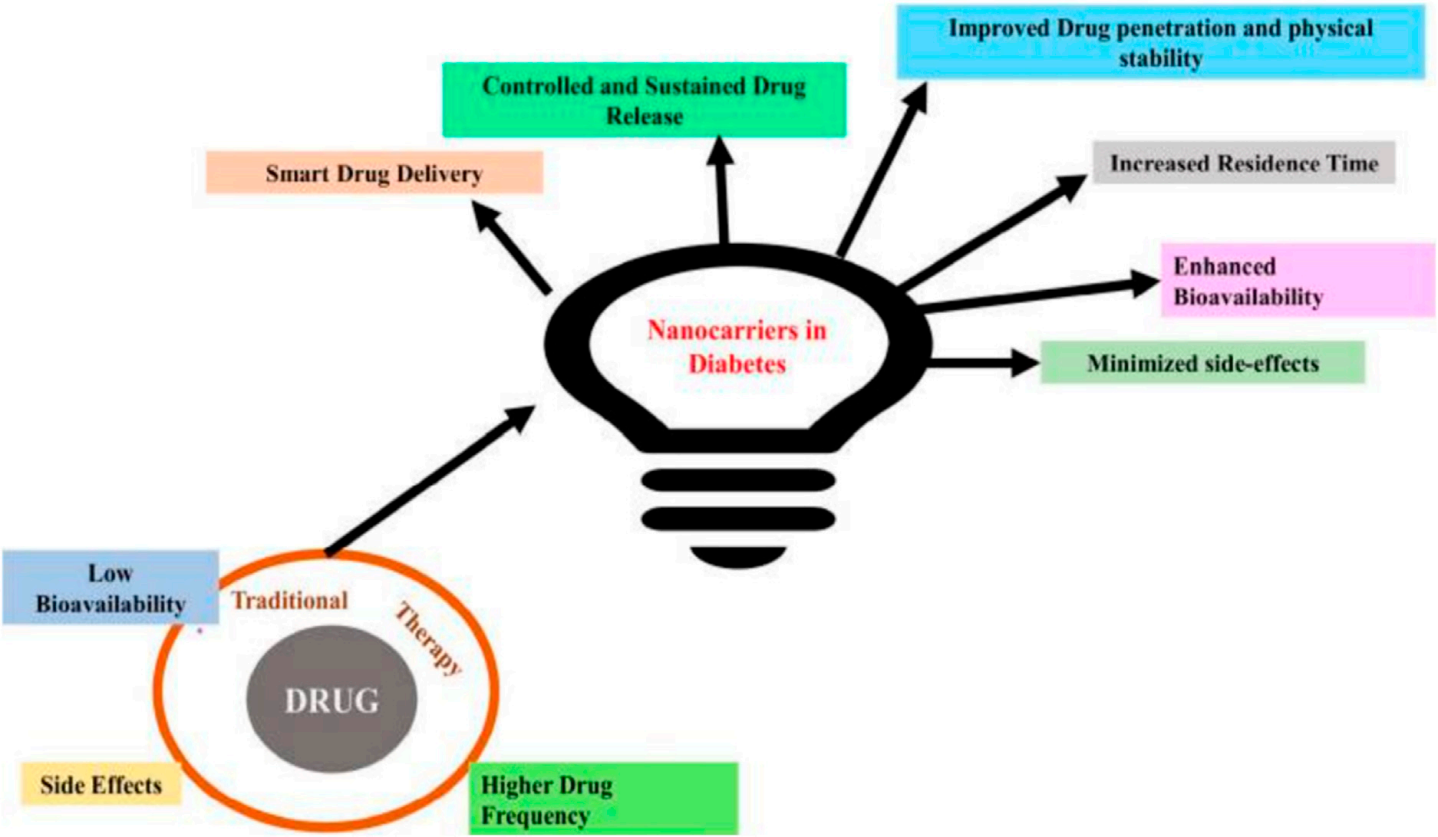
Demonstrates the various advantages of employing nanotechnology in diabetes management compared to traditional treatment methodologies. Reproduced with permission from Sarkhel et al. (2024).

Insulin is indispensable for managing T1DM and is often required in numerous instances of T2DM. The engineered characteristics of nanoparticles, such as toxicity control, stability, and drug release mechanisms, allow for the delivery of higher drug concentrations to targeted sites (Zaric et al., 2019). The capacity of nanoparticle systems to improve insulin delivery through targeted and controlled release mechanisms has attracted significant attention (Cheng et al., 2021; Karimi et al., 2016; Zhang et al., 2022). Nanocarriers present an innovative strategy by offering advantages such as enhanced drug stability and absorption, targeted delivery to specific tissues or cells, controlled or stimuli-responsive drug release, increased bioavailability, minimized side effects, and improved patient compliance (Figure 4). Sarkhel et al. have encapsulated the diverse applications of nanomaterials in diabetes management, emphasizing the distinctive attributes of nano-based drug delivery systems and intelligent drug delivery techniques (Sarkhel et al., 2024). These nanoparticles can be customized to react to physiological conditions, such as fluctuating glucose levels, thereby permitting a more personalized approach to insulin administration (Karimi et al., 2016; Sharmah et al., 2024). MSN-based nanocomposites have been used to deliver therapeutic molecules like insulin, GLP-1, exenatide, DPP-4 inhibitor and plasmid-containing GLP-1 genes for managing diabetes mellitus for the last decade (Sarkar et al., 2023). For instance, innovative systems have emerged that leverage glucose-responsive nanoparticles to release insulin during hyperglycemic episodes, thereby effectively imitating the pancreas’s physiological insulin secretion mechanism (Jeong et al., 2022; Volpatti et al., 2021).

Moreover, the inclusion of biocompatible materials in the formulation of nanoparticles ensures safety and efficacy in clinical applications (Tutty et al., 2022). Research has illustrated that nanoparticles can successfully encapsulate insulin, providing protection against degradation within the gastrointestinal tract during oral administration (Ren et al., 2023). This pioneering strategy not only enhances the stability of insulin but also promotes its absorption, yielding improved glycemic control in diabetic individuals. The integration of nanoparticles into insulin delivery systems indicates substantial potential for the development of more effective and patient-friendly diabetes treatments.

### 3.2 Delivery via transplantation of tissue-engineered islets

Tissue engineering has emerged as a groundbreaking technique in diabetes management, particularly in addressing the complications associated with the disease (Kaviani and Azarpira, 2016; Woo et al., 2023). This interdisciplinary domain merges biological, mechanical, and engineering principles to restore or enhance the functionality of damaged tissues and organs. Considering the increasing prevalence of diabetes and its complications, innovative strategies such as tissue engineering provide promising avenues for regeneration and repair, particularly in pancreatic and cellular contexts. Advancements within this field possess the potential to significantly enhance patient outcomes and offer alternatives to traditional therapies like insulin administration and organ transplantation.

#### 3.2.1 Pancreatic tissue engineering

**FIGURE 5:**
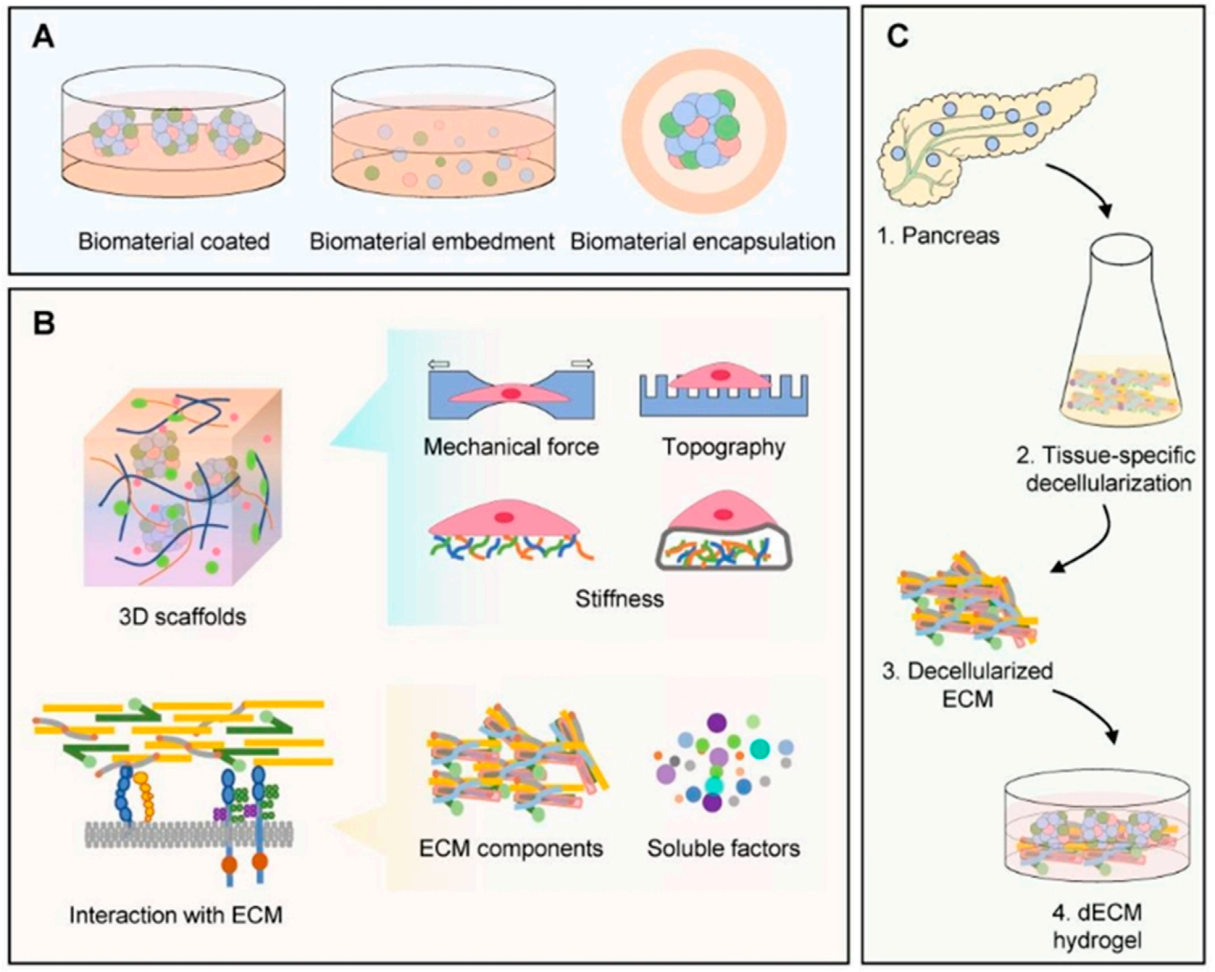
Illustrates a diagrammatic representation highlighting the application of materials in human islet organoids. **(A)** Applications of materials for production of human islet organoids, including strategies such as biomaterial coating, embedding, and encapsulation, plays a critical role in the advancement of diabetes treatments. **(B)** Biomaterials serve as three-dimensional scaffolds that replicate the native interactions with the extracellular matrix (ECM) essential for the generation of islet organoids. These scaffolds provide key factors such as mechanical forces, topographical features, stiffness, and signaling from ECM components and soluble factors. **(C)** The manufacturing process of decellularized ECM (dECM) materials is highlighted. This content is reproduced with permission from Jiang et al. (2022).

The domain of pancreatic tissue engineering is primarily focused on the creation of functional pancreatic tissues or bioartificial organs designed to restore insulin secretion in diabetic patients (Figure 5). Recent investigations have underscored the encouraging role of decellularized pancreatic scaffolds, which maintain the extracellular matrix (ECM) architecture and critical biochemical signals necessary for cell attachment and functionality. The application of decellularized pig pancreas has shown promise in establishing an optimal environment for insulin-producing cells, thereby addressing the impairment of beta-cell function in T1DM (Hao L. et al., 2024; Lim et al., 2023). Research indicates that these bioengineered tissues can effectively replicate the intrinsic architecture of the pancreas, which may enhance both the survival rates and functionality of transplanted islet cells (Lim et al., 2023). Furthermore, advancements in 3D bioprinting technology have enabled the fabrication of complex pancreatic structures, thereby improved vascularization and facilitating the delivery of essential nutrients required for maintaining cell viability (Soetedjo et al., 2021). Additionally, the incorporation of bioactive materials, such as silver nanoparticles, has demonstrated improved biocompatibility of these scaffolds, further supporting their clinical application (Qiu et al., 2022). In summary, pancreatic tissue engineering holds significant promise in the advancement of regenerative therapies for diabetes.

Cell transplantation, particularly the transplantation of islet cells, remains a fundamental aspect of T1DM management, with the primary objective of reinstating endogenous insulin production (Loretelli et al., 2020; Ramesh et al., 2013). However, barriers such as a limited supply of donors and the risk of immune rejection have hindered broader implementation. Recent advancements in tissue engineering have introduced innovative strategies aimed at enhancing the success rates of cell transplantation. For example, the application of interconnected toroidal hydrogels for islet encapsulation has proven effective in protecting transplanted cells from immune attacks. While still facilitating nutrient exchange (Ernst et al., 2019).

Additionally, the engineering of pluripotent stem cells into insulin-producing cells stands as a groundbreaking approach to generate a continual supply of functional cells for transplantation (Carvalho et al., 2022; Kasputis et al., 2018; Pagliuca et al., 2014). Further research has examined the potential of regulatory T cells that have been modified with insulin-specific chimeric antigen receptors to promote tolerance and reduce the risk of rejection during islet transplantation (Azad et al., 2024). These advancements in cell transplantation methodologies, when integrated with the principles of tissue engineering, hold the promise of significantly enhancing both the effectiveness and accessibility of diabetes treatments.

#### 3.2.2 Development of biomaterial scaffolds

The fabrication of biomaterial scaffolds constitutes a crucial aspect of tissue engineering within the framework of diabetes therapy, as they provide vital structural support for cellular growth and tissue regeneration. These scaffolds emulate the ECM and promote a three-dimensional structure that is conducive to cell proliferation, differentiation, and development. They may also be employed in the management of diabetic wounds, a common complication associated with diabetes (Tallapaneni et al., 2021). Scaffolds can be categorized into two main types based on their origin: natural and synthetic polymer-based scaffolds.

##### 3.2.2.1 Natural biomaterial scaffolds

**FIGURE 6:**
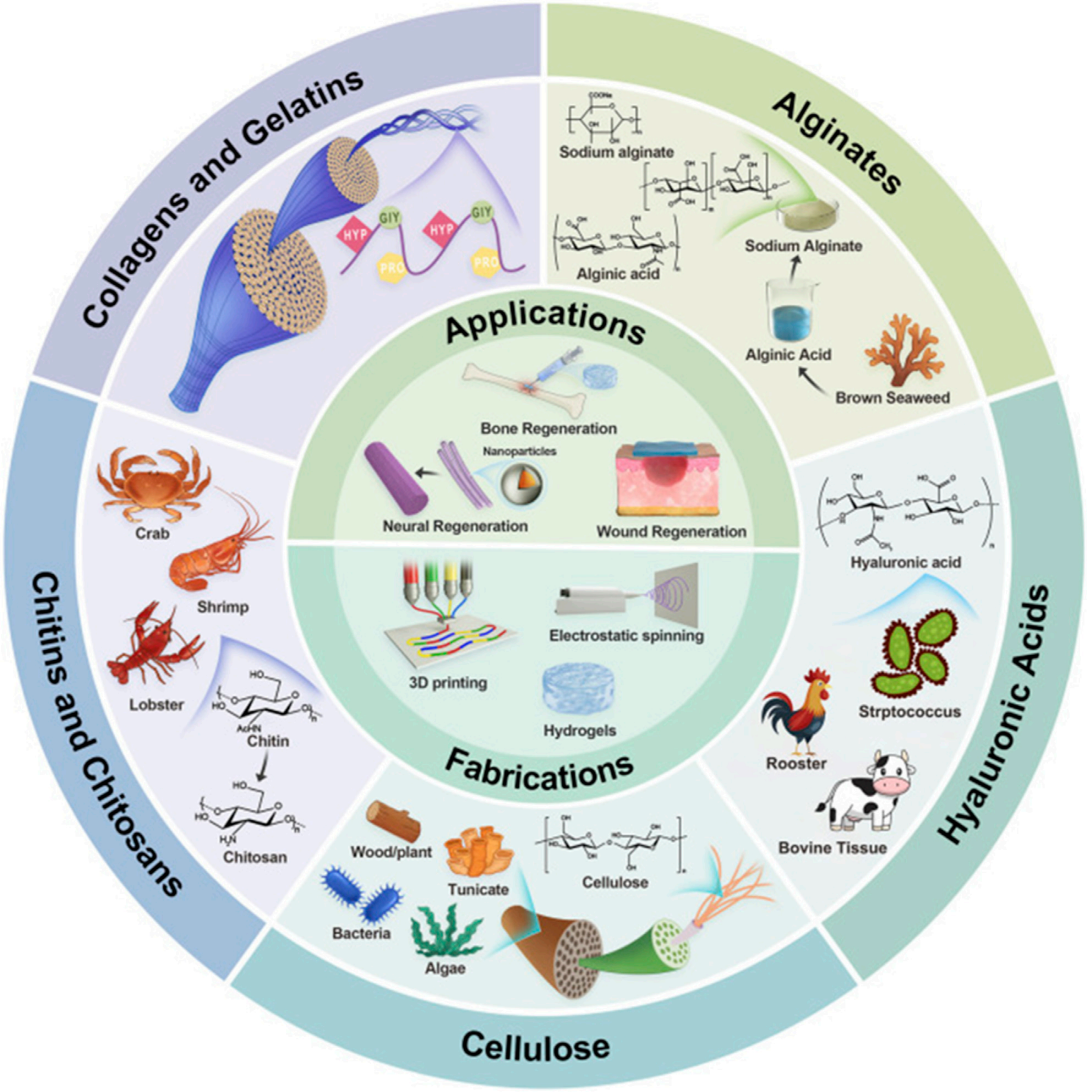
Illustrative schematic of naturally derived polymers:origin, structures, fabrications, and applications. Reproduced with permission from Hu et al. (2024a).

Natural biomaterials have garnered substantial interest in the field of tissue engineering because of their intrinsic biocompatibility and their capacity to facilitate cellular activities that are crucial for tissue regeneration (Bagheri et al., 2020; Mei et al., 2023). These materials, sourced from biological origins, include collagen, gelatine, chitosan, and alginate, which replicate the ECM of native tissues, thus fostering cellular interactions and enhancing healing processes (Naranda et al., 2021; Sonmezer et al., 2023). This is illustrated in Figure 6. For instance, collagen scaffolds are particularly recognized for their excellent properties regarding cell adhesion and biodegradability, rendering them suitable for applications in wound healing and regenerative medicine (Chu et al., 2018; Larijani et al., 2024). Chitosan, a natural polysaccharide, exhibits remarkable biocompatibility, biodegradability, and antimicrobial capabilities, positioning it as a promising candidate for wound healing and tissue engineering applications (Wang J. et al., 2024).

Moreover, the incorporation of bioactive molecules, such as growth factors and peptides, into natural materials can significantly enhance their regenerative capacity, leading to improved results in tissue repair and regeneration (Ravoor et al., 2021). Additionally, natural scaffolds can undergo modifications to improve their mechanical strength and degradation rates, thus allowing for customization tailored to specific applications. The inherent bioactive characteristics of natural biomaterials are further validated by their capacity to promote angiogenesis and facilitate tissue integration, both of which are essential for achieving favourable outcomes in tissue engineering (Goonoo, 2022).

The degradation behavior of these natural materials serves as a pivotal aspect concerning their application in biomedical contexts, significantly affecting their longevity, biocompatibility, and overall efficacy in tissue regeneration (Hu T. et al., 2024). Generally, natural materials are preferred due to their ability to undergo in vivo degradation, which permits a gradual replacement by newly synthesized tissue. Specifically, chitosan-based hydrogels have demonstrated a degradation process primarily governed by hydrolytic mechanisms, with degradation rates that can be modulated by varying the degree of crosslinking and the molecular weight (Lv et al., 2023). This characteristic proves particularly beneficial in scenarios such as drug delivery, where the establishment of controlled release profiles is vital for achieving therapeutic effectiveness.

The degradation byproducts of natural materials are frequently non-toxic and can be metabolically processed by the body, thereby minimizing the likelihood of adverse reactions (Xu et al., 2022). The capacity to engineer natural materials with specific degradation kinetics enhances their applicability across a range of uses, including bone regeneration, where it is optimal for scaffolds to degrade in synchrony with the formation of new bone (Koh et al., 2022). In summary, the degradation characteristics of natural materials not only contribute to their biocompatibility but also are integral to their functionality and efficacy in the field of regenerative medicine.

##### 3.2.2.2 Synthetic biomaterial scaffolds

Synthetic biomaterials, such as polycaprolactone (PCL), polylactic acid (PLA), and polyvinyl alcohol (PVA), have been engineered to address certain limitations associated with their natural counterparts (Deng et al., 2022). Research indicates that PCL scaffolds can effectively support the proliferation of mesenchymal stem cells and promote wound healing in models of diabetes (Abdollahi et al., 2024b). These synthetic materials provide customizable mechanical properties, controllable degradation rates, and can be fabricated into various forms, including fibers, films, and hydrogels (Lim et al., 2023; Li et al., 2020). This flexibility enables the optimization of material properties to better align with the mechanical characteristics of natural tissues, which is critical for applications involving implants and wound dressings. Investigations have shown that by adjusting the cross-linking density and the composition of the polymer network, researchers can develop hydrogels with tailored mechanical properties that are conducive to enhancing cell adhesion and proliferation in tissue engineering (Huang et al., 2023).

The adaptability of synthetic biomaterials facilitates the integration of bioactive agents, including growth factors or therapeutic drugs, allowing for their controlled release to foster healing and tissue regeneration (Guo et al., 2022b). Furthermore, the incorporation of nanomaterials into synthetic polymers has significantly improved their mechanical attributes, yielding materials that not only exhibit enhanced strength and durability but also demonstrate bioactivity that supports healing and integration with surrounding tissues (Abdollahi et al., 2024b). The integration of conductive materials within scaffolds has been explored to enhance the functional capacity of engineered tissues through improved electrical signalling, which is particularly important for insulin secretion in pancreatic cells (Wang and Jin, 2024). Moreover, advancements in three-dimensional printing technologies have facilitated the creation of intricate scaffold architectures that accurately replicate the structure of native tissues, thereby further augmenting the effectiveness of these biomaterials. The enhancement of integration and functionality in biomaterials has been highlighted by (Metwally et al., 2023). The adaptability of these mechanical properties is crucial for the effective incorporation of synthetic materials in clinical applications, as it enables the design of substances capable of enduring physiological stresses while supporting biological activities.

By amalgamating various functionalities within a single biomaterial, researchers are equipped to tackle diverse therapeutic challenges simultaneously. Such biomaterials can facilitate the controlled release of therapeutic agents, thereby promoting localized healing and reducing systemic side effects (Heidari et al., 2023). Additionally, these multifunctional materials can embed antibacterial characteristics to mitigate infections, which commonly arise in chronic wounds (Renuka et al., 2022).

Despite the considerable benefits offered by synthetic materials, significant concerns regarding their degradation and biocompatibility persist as critical hurdles in their utilization. For instance, materials engineered for temporary implants must degrade in synchronization with tissue healing to prevent complications linked to either premature breakdown or prolonged presence in the organism (Li et al., 2022). Moreover, ensuring the biocompatibility of synthetic materials is vital, as those that provoke adverse immune responses can incite chronic inflammation and result in implant failure (Ciatti et al., 2024; Kzhyshkowska et al., 2015). Recent progress has concentrated on the creation of biodegradable polymers that preserve their mechanical strength while systematically decomposing into non-toxic byproducts (Guo et al., 2022b). Addressing these concerns surrounding degradation and biocompatibility is essential for the successful transition of synthetic materials from laboratory settings to clinical implementations, guaranteeing that they offer safe and effective solutions for patients.

### 3.3 Delivery by transdermal delivery

**FIGURE 7:**
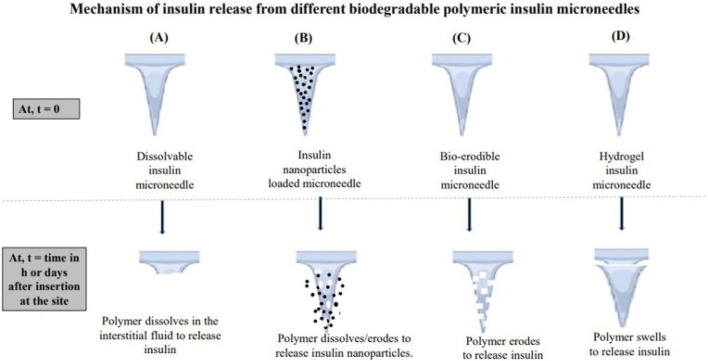
Mechanism of insulin release from the microneedles. **(A)** shows a soluble insulin microneedle that releases insulin through polymer dissolution. **(B)** shows a microneedle loaded with insulin nanoparticles, and insulin is released through the biodegradation of the shell or matrix. **(C)** shows a biodegradable insulin microneedle, and insulin is released after enzymatic hydrolysis. **(D)** shows a hydrogel insulin microneedle that continuously releases insulin after entering the dermis. Reproduced with permission from Starlin et al. (2024).

**FIGURE 8:**
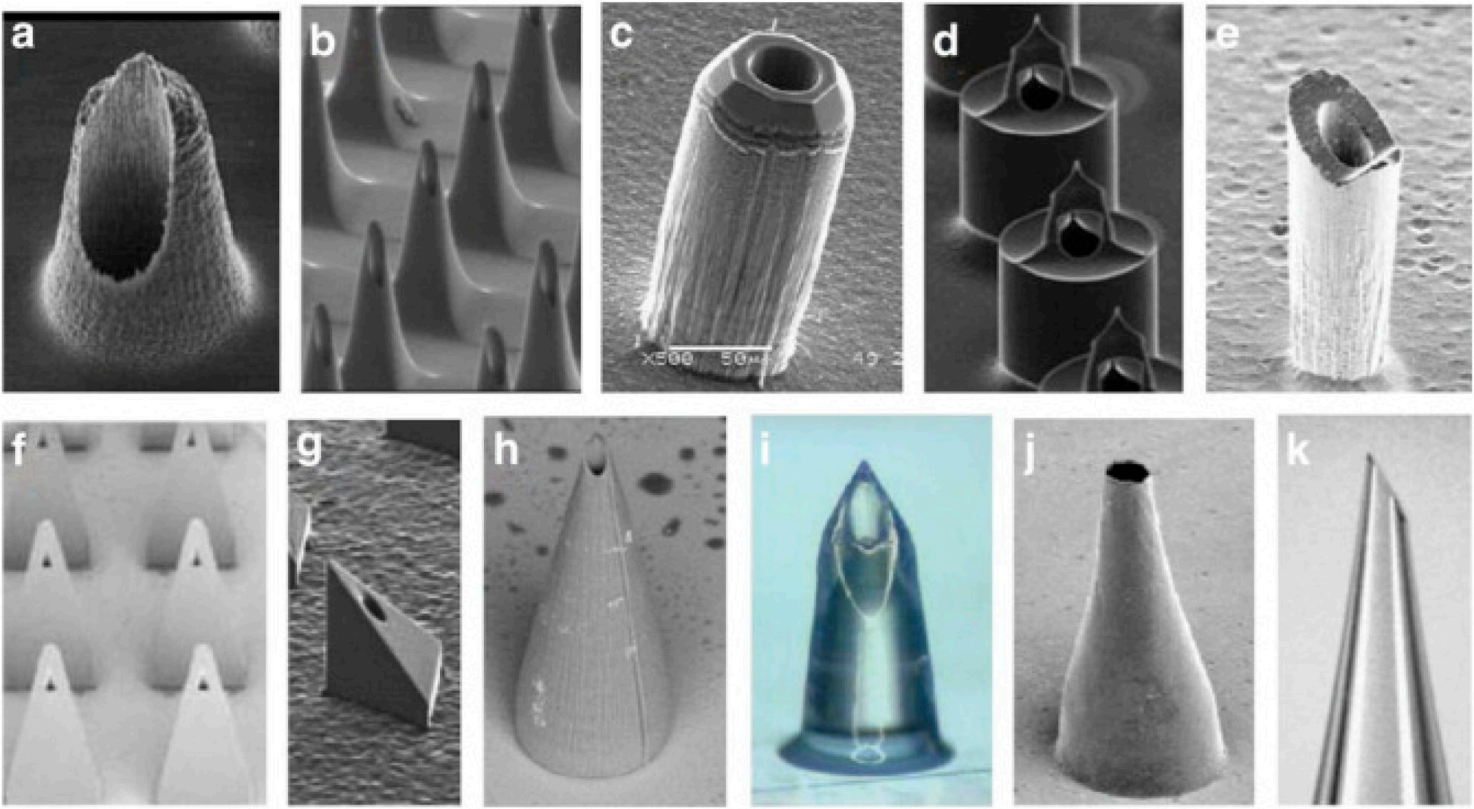
Hollow microneedles created from silicon and polymers. **(A, B)** Hollow microneedles with a tapered shape. Mukerjee et al. (2015), Wilke et al. (2005) **(C)** Hollow silicon microneedles with sharp tips Ma et al. (2006). **(D)** cylindrical microneedles with a side-opening orifice Zhang et al. (2009) **(E)** Hollow silicon microneedles with sharp tips Baron et al. (2008) **(F)** Hollow microneedles by exposing X-ray through a mask onto PolyMethylMetaAcrylic. Moon et al. (2005) **(G)** A micro-gear pump Amirouche et al. (2009) **(H)** Microneedles with on-board fluid pumps Lin and Pisano (1999) **(I)** Flow of liquid through glass hollow microneedles controlled by CO2 gas pressure Martanto et al. (2006) **(J)** An electrical microneedle applicator Verbaan et al. (2008) **(K)** Flow of liquid through hollow microneedles controlled by a syringe pump Gupta et al. (2009). Reproduced with permission from Kim et al. (2012).

Microneedle technology has emerged as a groundbreaking approach for insulin delivery, providing a minimally invasive alternative to conventional injection techniques (Bigham et al., 2025; Hong et al., 2022; Zong et al., 2022). The mechanism of insulin release from microneedles is depicted in Figure 7. These micro-scaled needles, which typically range in length from 25 to 1,000 μm (Figure 8), can penetrate the outer layer of the skin while circumventing nerve endings, thus minimizing discomfort and pain for patients (Chen et al., 2019; Luo et al., 2023; Wang et al., 2020). Recent advancements in the manufacturing technologies for microneedles, including 3D printing and mold-based methods, have enabled the creation of arrays capable of delivering precise dosages of insulin (Razzaghi et al., 2024). Evidence suggests that these microneedle arrays achieve bioavailability levels that are comparable to those obtained from traditional subcutaneous injections while significantly enhancing patient adherence due to their ease of use and reduced pain perception (Li et al., 2022; Queiroz et al., 2020). Furthermore, the incorporation of biodegradable materials in microneedle design has allowed for sustained insulin release, presenting a viable solution for long-term diabetes management (Chakraborty et al., 2023; Rajput et al., 2021). A nanoparticle-loaded microneedle (MN) patch, designed for transdermal drug delivery, aims to achieve blood glucose control and reactive oxygen species (ROS) scavenging for the synergistic treatment of diabetic nephropathy, thereby enhancing the efficiency of transdermal drug delivery while extending the duration of insulin action (Zheng et al., 2025). In summary, microneedle technology stands as a promising strategy for advancing insulin delivery systems and subsequently enhancing the quality of life for individuals with diabetes.

### 3.4 Smart delivery systems for diabetes management and treatment

The Smart delivery systems represent the cutting edge of insulin administration technology, merging innovative biomaterials with responsive mechanisms to develop dynamic delivery platforms. These systems are engineered to release insulin in a controlled manner, guided by real-time blood glucose monitoring, thereby providing a customized approach to managing T1DM (Condren et al., 2019; Latham, 2019; Moser et al., 2025; Renard, 2023). For example, hydrogels that expand or contract in response to changes in glucose concentrations have been developed, enabling on-demand insulin release as required (Ali et al., 2022; Annicchiarico et al., 2024). Furthermore, the incorporation of wearable technology within these smart delivery systems facilitates continuous glucose monitoring, which allows for automatic insulin administration in reaction to fluctuations in glucose levels (Renzu et al., 2024). This heightened level of responsiveness not only improves glycemic control but also reduces the risk of hypoglycemia, a prevalent issue in diabetes management. As research progresses, the potential for intelligent delivery systems to transform insulin therapy becomes increasingly evident, paving the way for more effective and user-friendly diabetes care solutions.

## 4 The role of biomaterials in diabetic wound healing

**FIGURE 9:**
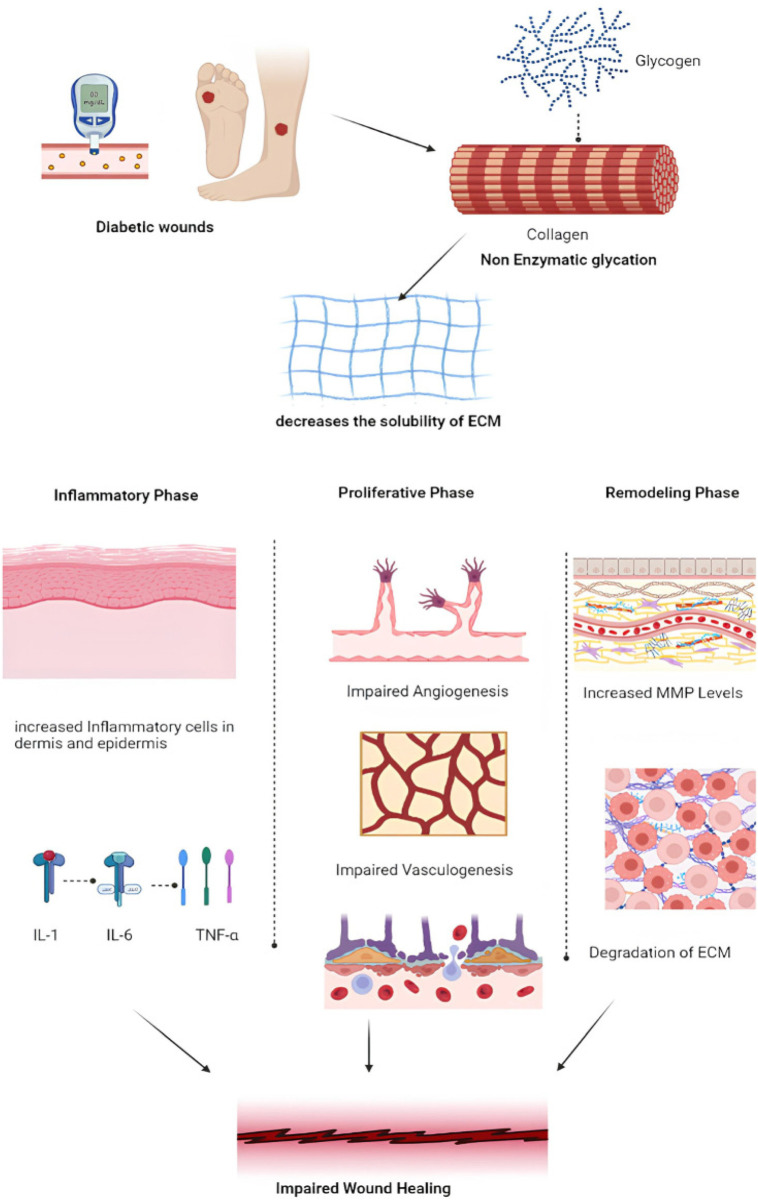
Schematic diagram of diabetic wound repair. Reproduced with permission from Aldahish et al. (2024).

Current practices in managing diabetic wounds are based on four essential principles: (1) debridement, (2) infection control, (3) offloading, and (4) revascularization (Hu et al., 2022). In the context of diabetic wounds, particularly foot ulcers, the primary factor contributing to delayed healing is the diminished synthesis of collagen. This reduction adversely affects the solubility of the extracellular matrix (ECM) and provokes an exaggerated inflammatory response (Nirenjen et al., 2023). The inflammatory phase is marked by the secretion of pro-inflammatory cytokines such as IL-1, IL-6, and TNF-α. The subsequent proliferative phase is characterized by impaired angiogenesis and vasculogenesis, whereas in the remodeling phase, an increase in matrix metalloproteinases (MMPs) results in further degradation of the ECM, thereby exacerbating the challenges associated with wound healing (Figure 9). These factors present considerable hurdles for clinical management. Although traditional dressings have historically been essential in wound care, their effectiveness in treating diabetic wounds is significantly limited (Saco et al., 2016; Wang et al., 2024).

### 4.1 Limitations of traditional dressings for diabetic wound treatment

Diabetic wounds exhibit a complex pathophysiological profile that includes impaired angiogenesis, a weakened immune response, and an increased vulnerability to infections (Rodriguez-Rodriguez et al., 2022). A significant limitation of conventional dressings, including gauze and hydrogels, is their singular functionality, which fails to adequately address the diverse challenges associated with diabetic wounds (Venkatesan and Rangasamy, 2023; Zhang et al., 2023a). These traditional dressings often lack the incorporation of bioactive agents that could facilitate healing, and their capacity to prevent bacterial proliferation is insufficient, leading to a heightened risk of infection (Zhou et al., 2024a). Furthermore, issues with adherence and retention of these dressings can necessitate frequent changes, which may disrupt the healing process and inflict additional pain and discomfort on patients (Jiang et al., 2023).

**FIGURE 10:**
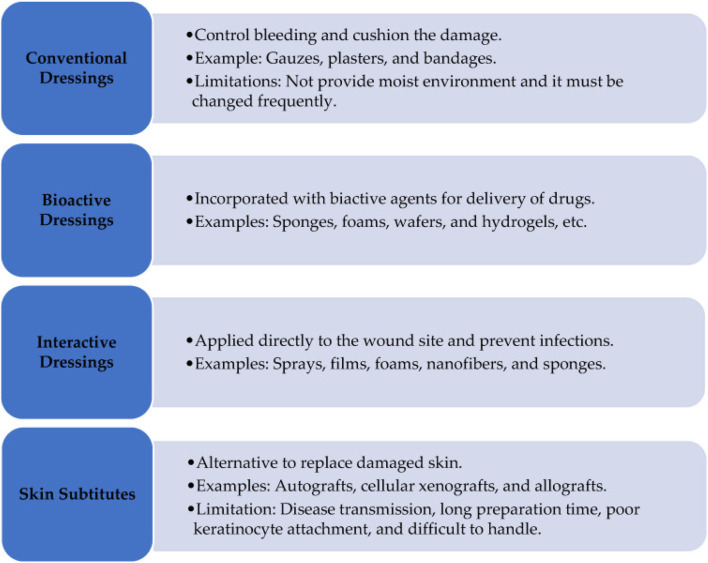
Classification of wound dressings. Reproduced with permission from Alven et al. (2020).

Moreover, the healing duration associated with conventional dressings can be extended, raising concerns for diabetic individuals who are predisposed to complications such as foot ulcers and potential amputations (Andrews et al., 2015; Sahu et al., 2018). The absence of advanced features in these dressings means they do not support critical physiological processes, such as angiogenesis and collagen deposition, which are essential for effective wound repair (Zhang et al., 2024). Consequently, there exists a pressing need for the formulation of more effective wound care solutions that integrate bioactive materials along with multifunctional attributes to enhance the healing of diabetic wounds (Cai F. et al., 2023). Various types of dressings, including conventional, bioactive, and interactive dressings, as well as skin substitutes, are being employed to treat wounds (Alven et al., 2020) (Figure 10).

In conclusion, while traditional dressings have played a crucial role in wound management, their inadequacies in addressing diabetic wounds underscore the necessity for a transition towards more innovative treatment strategies that can effectively tackle the distinct challenges they present. The diabetes patients can benefit significantly from the incorporation of sophisticated biomaterials and innovative technologies, which may prove instrumental in addressing existing challenges and enhancing patient outcomes in the management of diabetic wounds (refer to Table 3).

**TABLE 3:** A selection of biomaterial products for clinical management of diabetic wounds.

Product name	Main features	Clinical application status	Treatment Stage	References
Codfish Skin (After Decellularization and Freeze-drying Treatment)	1.Excellent biocompatibility 2.Abundant in collagen and growth factors 3.Natural antibacterial properties 4.Economical and readily available	In the Odinn Phase III clinical trial	Diabetic foot ulcer complication stage, especially suitable for deep diabetic foot ulcers (UT grade 2 or 3), that is, ulcers penetrating to the tendon or joint capsule (UT grade 2) or deep into the bone or joint (UT grade 3)	Esmaeili et al. (2023), Pei et al. (2023)
“Subiyi^®^” Xianglei Diabetic Foot Ointment	1.Modulates macrophage activity 2.Enhances inflammation resolution and promotes tissue repair 3.Reconfigures the wound microenvironment	Utilized in Taiwan (China), Singapore, Malaysia, etc.,; has received Fast Track Certification from the US FDA	Diabetic foot ulcer complication stage, applicable to patients with Wagner grade 1 diabetic foot ulcers and wound cross-sectional area less than 25 cm^2^	(Chinese Society of Endocrinology, 2024)
Chitosan Pressure Ulcer and Diabetic Foot Dressing	1.Forms an artificial skin for protection and enhancement of wound healing 2.Free from antibiotics, analgesics, or anesthetics 3.No delayed hypersensitivity reactions or irritation to skin and mucous membranes	Aids in alleviating edema, pain, ulceration, and other complications associated with Stage I, II, and III pressure ulcers and diabetic foot ulcers	Diabetic foot ulcer complication stage, can be used to relieve complications such as stage I, II, and III pressure ulcers and diabetic foot ulcers	(Huang et al., 2023; Mirbagheri et al., 2023; Ren et al., 2022)
Mandabang Diabetic Foot Wound Cleaning Liquid Dressing	1.Highly efficient sterilization with a safety profile 2.Broad antibacterial spectrum 3.Acts within 15–30 s, achieving a 99.9999% kill rate 4.Exhibits antibacterial properties even at minimal concentrations	Applicable for wound care and debridement across various departments	Diabetic foot ulcer complication stage	From the internet (http://www.chinamsr.com/2021/0108/116520.shtml)
Recombinant Lysozyme-Antibacterial Peptide Fusion Protein	1.Secrete growth factors to achieve self-repair 2.Accelerate the formation of micro vessels 3.Exhibits low toxicity and minimal irritation 4.no inflammatory exudation	Under research	Diabetic complication stage	Chen et al. (2018)
Hairun Biology Anti-Infection Dressings	1.Exhibits anti-infection capabilities 2.Reduces dressing change frequency	Widely implemented in the clinical management of diabetic foot ulcers and other wound surfaces	Diabetic foot ulcer complication stage	From the internet (https://ylqx.qgyyzs.net/user/web7105/)
Recombinant Growth Factor Gel	1.Promotes wound healing 2.Aids in expediting the repair of diabetic foot ulcers	Extensively employed in the adjunctive treatment of wound surface repairs, including diabetic foot ulcers	Diabetic foot ulcer complication stage	Das et al. (2023)
PDA@Ag/SerMA microneedles	1.safe, effective, painless and minimally invasive medication administration through the skin 2.promote cell mitosis 3.accelerate wound healing 4. The wound healing rate of mice reached 95% within 12 days 5.approximately 100% antimicrobial efficacy against *Staphylococcus aureus* and *Escherichia coli* under 808 nm near-infrared irradiation	Only in mice	Diabetic foot ulcer complication stage	Chen et al. (2024d)

### 4.2 The utilization of innovative wound dressings in diabetic wound healing

**FIGURE 11:**
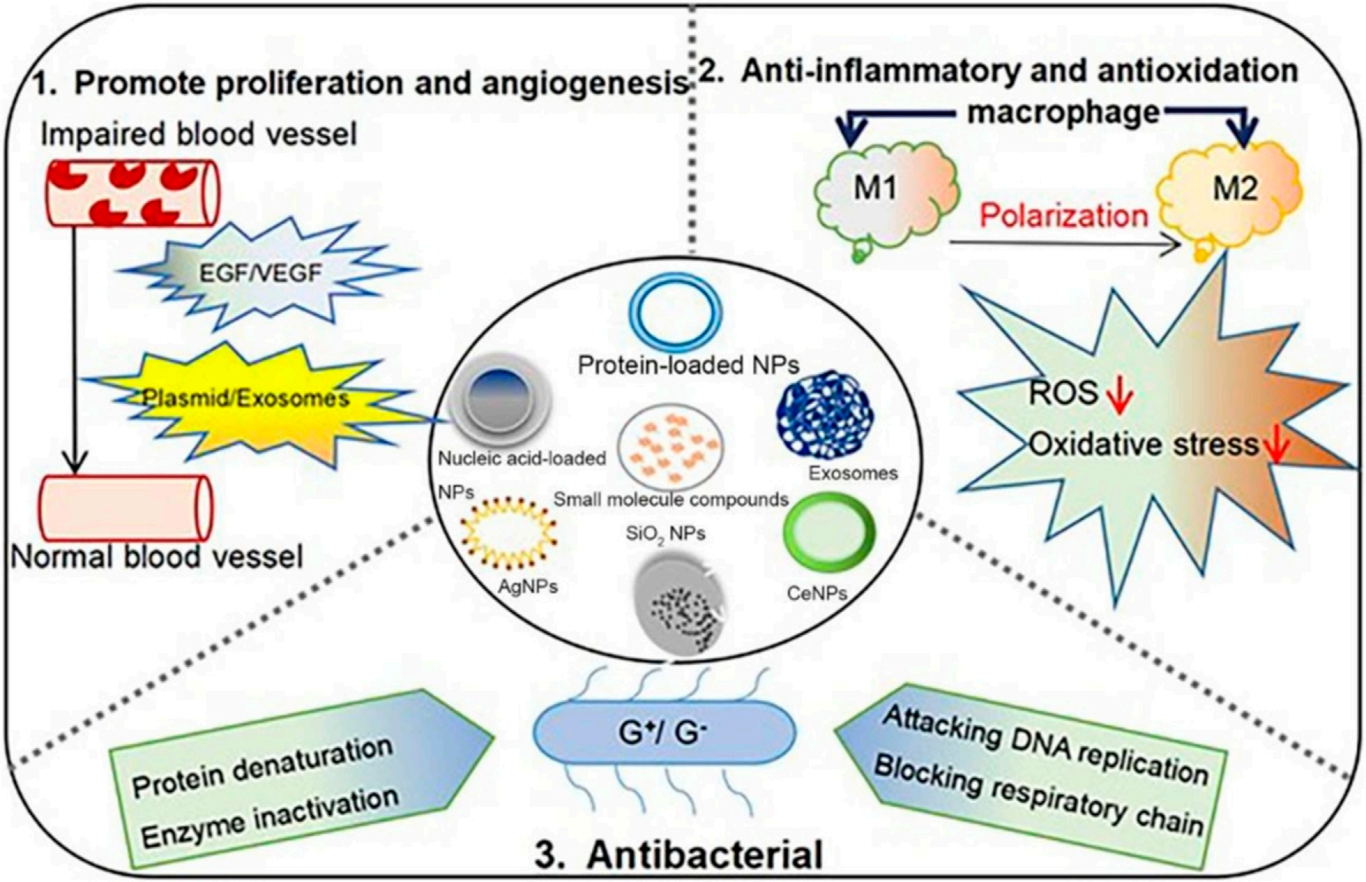
Presents a diagrammatic representation illustrating the various classifications and therapeutic mechanisms associated with biomaterials utilized in the management of diabetic wounds. This illustration is reproduced with permission from Qin et al. (2022).

Recent innovations in wound dressing technologies have culminated in the creation of multifunctional dressings that incorporate biocompatible materials along with bioactive agents (Figure 11). According to a systematic review conducted by Vargas et al., bioactive glass (BG)-based materials show promise in expediting all phases of diabetic wound healing and improving the overall quality of wound recovery (Vargas et al., 2024). For example, electrospun nanofibers and hydrogels are employed to fabricate dressings that not only provide a protective barrier but also deliver therapeutic agents directly to the wound site (Fahimirad and Ajalloueian, 2019; Hong et al., 2023; Yang and Xu, 2023). Furthermore, the integration of electrical stimulation within wound dressings has revealed potential for enhancing healing rates by fostering cellular activities and optimizing blood circulation to the affected regions (Asadi and Torkaman, 2014; Fan et al., 2024; Hu Y. W. et al., 2024). Additionally, the incorporation of antimicrobial additives into wound dressings is increasingly gaining traction, providing an additional layer of defense against infections, which are a common complication in diabetic wounds (Chen et al., 2024d; Firoozbahr et al., 2023; Li et al., 2024; Rozman et al., 2020). In summary, advancements in wound dressing technologies signify a considerable leap forward in the effective management of diabetic wounds, offering tailored solutions.

#### 4.2.1 Biocompatible materials

The biocompatibility of materials is a crucial factor in the development of biomaterials for biomedical applications, particularly in the context of diabetic wound healing (Nandhakumar et al., 2022; Ren et al., 2022; Xu et al., 2023). These materials are specifically designed to interact positively with biological systems, thereby reducing adverse reactions while facilitating healing processes (Chan et al., 2023; Naahidi et al., 2017). Recent studies have underscored the promising potential of various biocompatible materials, such as chitosan, alginate, and hyaluronic acid, which have shown encouraging outcomes in promoting the healing of diabetic wounds (Peng et al., 2022). Although clinical trials remain limited, chitosan has emerged as a highly effective alternative for modulating local inflammatory responses and promoting wound healing, especially in patients with comorbid conditions that hinder typical skin healing processes, such as diabetes and vascular insufficiency (Maita et al., 2022). Chitosan-based biomaterials have gained recognition for their efficacy in wound healing, characterized by their antibacterial properties and ability to enhance cellular proliferation, rendering them suitable candidates for applications in wound care (Cai and Li, 2020; Rajinikanth et al., 2024). Systematic reviews and meta-analyses have established that, relative to the standard of care (SOC), patients receiving placenta-derived biomaterial treatments demonstrate a superior rate of complete wound healing in cases of diabetic foot ulcers (DFUs) (Ruiz-Munoz et al., 2024). Chen et al. corroborated that placenta-based tissue products exhibited the highest likelihood of wound healing (p-score = 0.90), followed by living cell skin substitutes (p-score = 0.70), acellular skin substitutes (p-score = 0.56), and advanced topical dressings (p-score = 0.34) when measured against standard DFU care (Chen L. et al., 2024).

**FIGURE 12:**
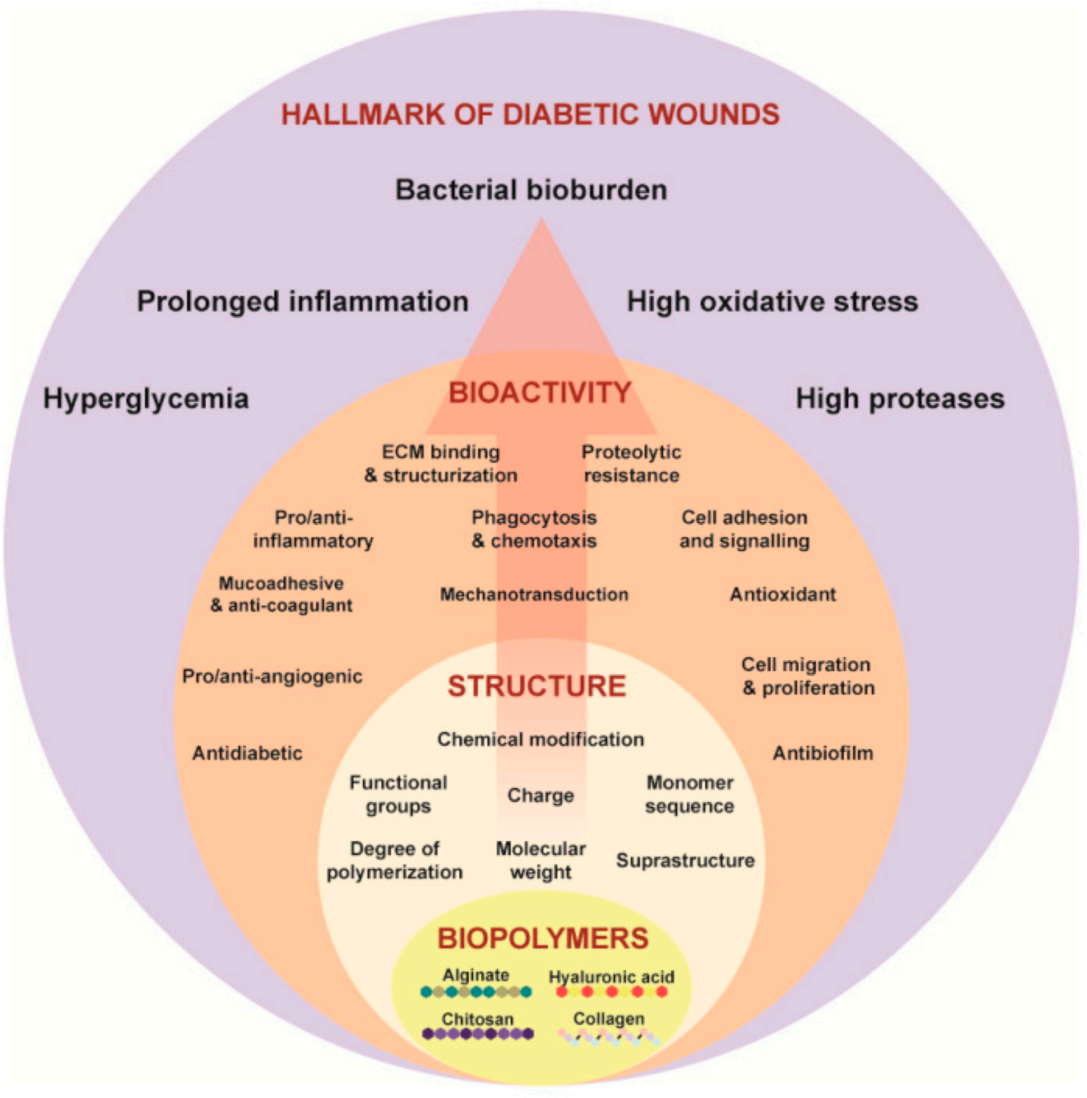
Illustrates the structure–function paradigm as represented in Equation concerning biopolymers such as alginate, chitosan, hyaluronic acid, and collagen, which target the critical features of chronic wounds. Reproduced with permission from Sharma and Kishen (2024).

Angiogenesis and cellular migration are fundamental processes in wound healing, which are frequently disrupted in diabetic wounds (Yang et al., 2024). Consequently, an optimal biomaterial should facilitate the development of new blood vessels to enhance blood flow and oxygen supply at the wound site. Achieving these characteristics necessitates the engineering of physico-chemical properties at both chemical and molecular levels, ensuring alignment with the required bioactivity for wound healing in diabetic conditions (Sharma and Kishen, 2024) (Figure 12). This necessity highlights the importance of comprehending the structure–function relationship within biopolymers.

Moreover, integrating natural compounds into these materials can enhance their biocompatibility and therapeutic efficacy, as demonstrated by the incorporation of honey and plant extracts in wound dressings (Prasathkumar and Sadhasivam, 2021; Yasin et al., 2023). The advancement of nanomaterials also presents novel opportunities for improving biocompatibility and functionality (Barhoum et al., 2022). Research has shown that these materials can enhance cellular responses and tissue integration (Bai et al., 2020). Overall, the creation of biocompatible materials is crucial for developing effective treatments for diabetic wounds, ensuring that they not only promote healing but also seamlessly integrate with the body’s biological systems.

#### 4.2.2 Bioactive molecules

**FIGURE 13:**
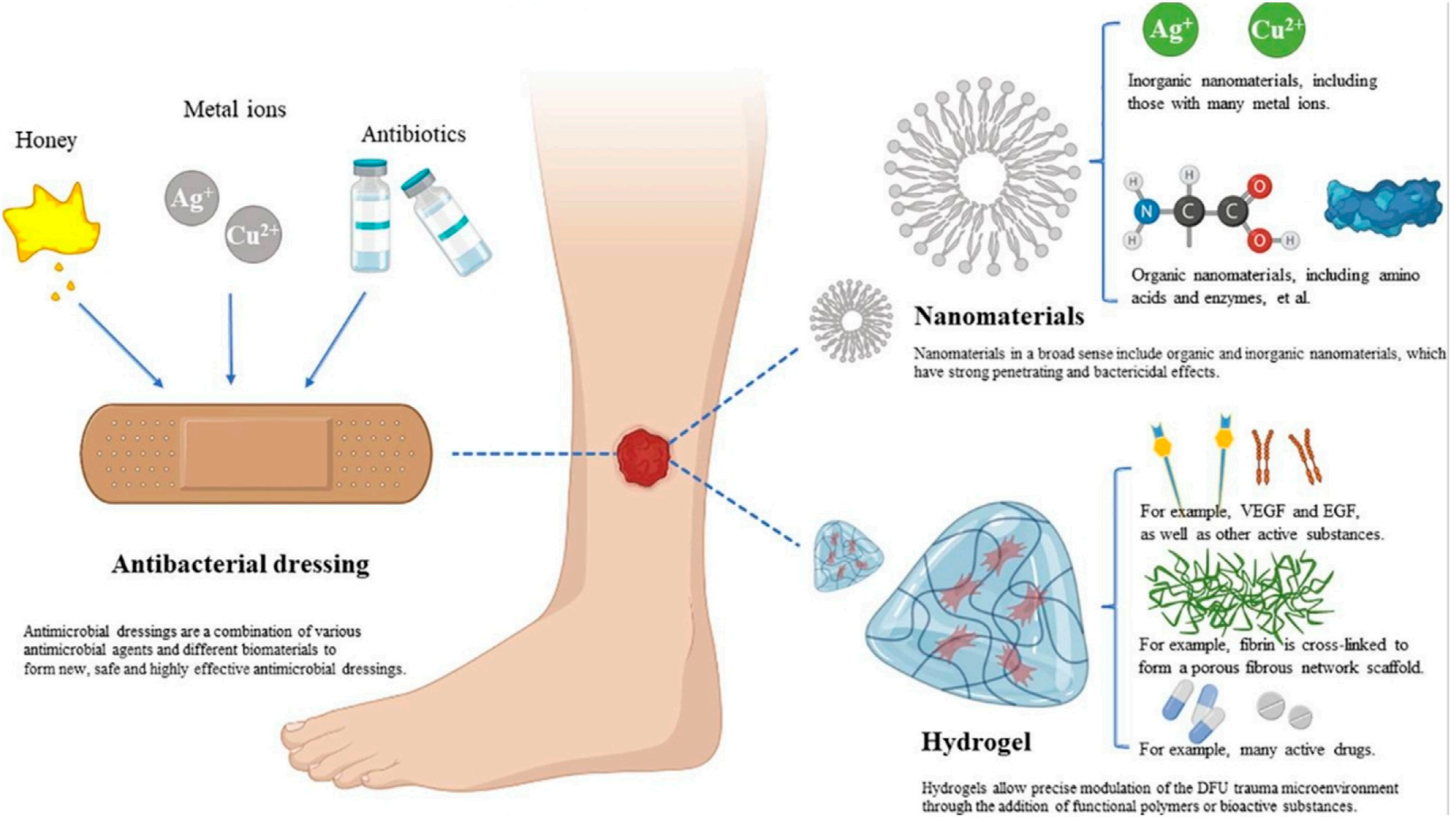
A visual representation illustrating antibacterial dressings, nanodressings, and hydrogel dressings. Reproduced with permission from Jiang et al. (2023).

Bioactive molecules play a critical role in the wound healing process, especially for individuals with diabetes, where natural healing mechanisms are often hindered (Moses et al., 2023; Oprita et al., 2023; Sultana et al., 2024). These molecules can be integrated into biomaterials to bolster their therapeutic effects. For instance, growth factors, cytokines, and antimicrobial peptides are currently being studied for their capacity to stimulate essential cellular activities, including migration, proliferation, and angiogenesis (Takahashi et al., 2021; Umehara et al., 2022; Yue et al., 2022). Recent research indicates that the incorporation of bioactive molecules into hydrogels and scaffolds can markedly improve healing outcomes for chronic wounds (Chen et al., 2024b; Rathna and Kulandhaivel, 2024; Yusuf and Adeleke, 2023). Figure 13 provides a schematic representation of various biomaterial dressings. Additionally, studies have highlighted the potential of metal nanoparticles as bioactive agents in diabetic wound therapy, offering antimicrobial properties while facilitating tissue regeneration (Zheng et al., 2024). The development of intelligent biomaterials capable of controlling the release of these bioactive molecules represents a promising research area, enabling targeted delivery and enhanced therapeutic effectiveness (Huang et al., 2023). Therefore, the strategic incorporation of bioactive molecules alongside biomaterials marks a significant advancement in diabetic wound treatment, fostering a more effective healing process.

## 5 Challenges and future directions

The utilization of biomaterials in diabetes management represents a promising Frontier with significant potential, particularly in the domains of diabetes treatment and wound healing. Nonetheless, the deployment of biomaterials for diabetes management, especially in the context of wound healing, embodies a dual-edged sword characterized by both benefits and drawbacks.

From a positive perspective, biomaterials such as hydrogels, nanoparticles, and scaffolds present enhanced characteristics that can markedly improve outcomes in wound healing (Fadilah et al., 2022; Leng et al., 2022; Zhang Z. et al., 2024). These biomaterials can be meticulously engineered to facilitate controlled drug release, encourage angiogenesis, and amplify cellular responses, effectively addressing the complex, multifactorial nature of diabetic wounds. The integration of bioactive agents, including growth factors and exosomes derived from stem cells, into these materials has the potential to further stimulate tissue regeneration and enhance healing rates (Jing et al., 2023). Furthermore, biomaterials can be customized to exhibit antibacterial properties, thereby diminishing the risk of infection, a frequent complication associated with diabetic wounds (Zheng et al., 2024).

Conversely, the application of biomaterials is not devoid of challenges. A notable concern is the risk of immune rejection or adverse reactions, particularly in relation to synthetic materials (Tripathi et al., 2023). The biocompatibility of these materials is a critical aspect that necessitates thorough evaluation to prevent complications that could impede rather than promote healing (Zhao et al., 2023). Additionally, the intricate environment of diabetic wounds may hinder the effective performance of biomaterials. Elevated levels of reactive oxygen species (ROS) within diabetic wounds can undermine the efficacy of specific biomaterials, highlighting the need for the development of advanced formulations capable of alleviating oxidative stress (Cai et al., 2023a; He et al., 2023; Yao et al., 2019). Moreover, the cost and accessibility of sophisticated biomaterials may present an obstacle to their wide-scale adoption in clinical settings (Ansari and Darvishi, 2024; Chen and Liu, 2016). While these materials exhibit considerable promise, their incorporation into standard diabetes management requires a meticulous assessment of their long-term effects, potential complications, and overall cost-effectiveness.

In summary, although biomaterials present exciting prospects for improving diabetes management and wound healing, it remains imperative to weigh their benefits against potential drawbacks. Critical factors regarding biocompatibility, safety profiles, and long-term efficacy of biomaterials necessitate further exploration to guarantee their safe integration into clinical practice. Ongoing research and clinical trials will play a vital role in identifying the most effective and safe applications of biomaterials in this context, ultimately striving to enhance patient outcomes in diabetes care.

### 5.1 Safety and efficacy of biomaterials

The safety and efficacy of biomaterials are of paramount concern as their use in clinical applications continues to expand (Kantak and Bharate, 2022). These materials must engage positively with biological systems, facilitating healing while minimizing adverse reactions (Knopf-Marques et al., 2016). The challenge lies in ensuring that these biomaterials do not provoke toxic responses or incite chronic inflammation, which could compromise their intended function. Advances in the understanding of the interaction between biomaterials and the immune system have paved the way for the design of materials capable of favorably modulating immune responses, thereby enhancing their therapeutic potential (Salthouse et al., 2023). Furthermore, the advancement of nanotoxicity evaluations is essential, as nanoparticles employed in biomaterials may pose risks distinct from their bulk forms (Akcan et al., 2020). As this discipline progresses, it is imperative for researchers to prioritize the creation of standardized protocols for assessing the safety of biomaterials to streamline regulatory approval processes and enhance clinical translation (Josyula et al., 2021).

In the last decade, a significant concentration of clinical research on biomaterials has emerged, closely linked to advances in fundamental research. Nonetheless, the findings derived from basic research may not necessarily translate directly to human applications (Socci et al., 2023). As previously noted in this manuscript, the biocompatibility and efficacy of certain established biodegradable biomaterials have been validated through clinical trials (Arrizabalaga and Nollert, 2018). The academic community broadly recognizes the potential for biomaterials to be integrated with agents such as stem cells and bioactive factors (Wilems et al., 2019). However, challenges such as ethical considerations and the variability in source materials hinder seamless clinical translation. Moreover, most animal models utilized in fundamental research are rodents, which, while advantageous due to their availability and established modeling techniques, present a significant limitation: their wound-healing mechanisms differ from those in humans (Nuutila et al., 2016). Several clinical trials have yet to achieve the anticipated outcomes in human subjects, causing stagnation in clinical translation efforts (Shamshad et al., 2023).

Consequently, it is crucial for basic research teams to foster close collaboration with clinical departments. By aligning with genuine clinical needs, they should conduct focused basic research aimed at facilitating clinical translation, thereby identifying safer and more effective biomaterials for application in clinical settings.

### 5.2 Possibility of personalized treatments

The capacity for real-time monitoring and data analysis marks a significant evolution in the domain of biosensors. With the progression of data analytics and machine learning, the interpretation of biosensor data has become increasingly sophisticated, enabling predictive insights and tailored healthcare solutions (Childs et al., 2024; Zhang et al., 2021; Schackart and Yoon, 2021). Real-time health monitoring systems can amalgamate data from various biosensors, offering a holistic view of a patient’s health status (Paganelli et al., 2022; Wu et al., 2023; Li et al., 2021). This integration allows for timely interventions and enhanced management of chronic ailments, including diabetes and cardiovascular conditions. Additionally, the emergence of mobile applications that connect with biosensors empowers patients to conveniently monitor their health metrics, thus promoting greater involvement in their own care (Gecili et al., 2020). The future of biosensors is poised to enhance patient outcomes and healthcare efficiency through the provision of actionable insights derived from real-time data analysis.

**FIGURE 14:**
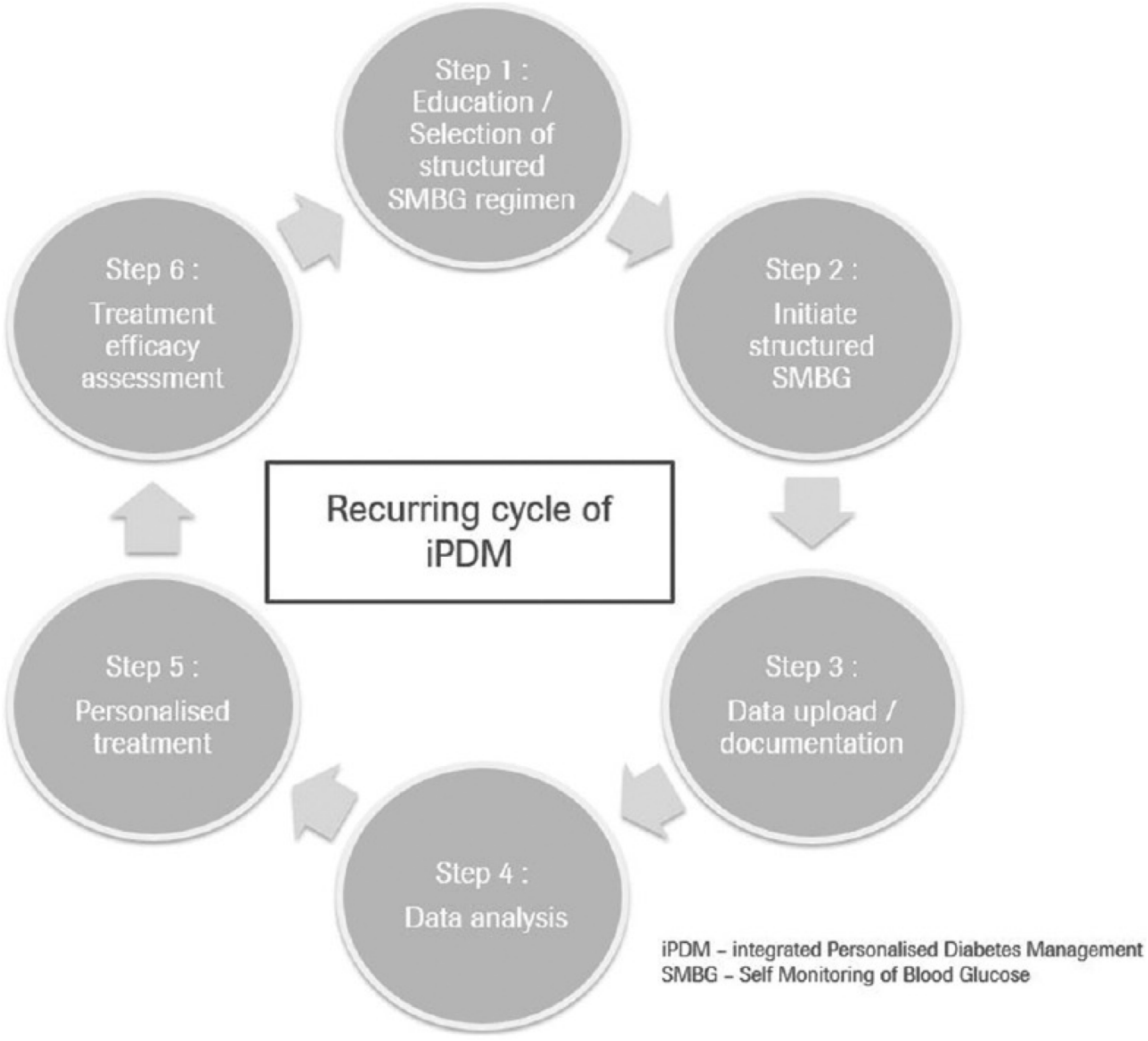
Depicts the Cycle of Integrated Personalized Diabetes Management, which comprises six iterative steps and forms a continuous revolving circle, applicable to each patient over differing timeframes. Reproduced with permission from Kalra et al. (2022).

The shift towards personalized medicine signifies a groundbreaking approach within healthcare, particularly regarding biomaterials. Individual patients exhibit variability in their financial resources and a range of personal factors. A systematic analysis conducted by Maria et al. revealed no statistically significant differences in HbA1c values among patients with type 1, type 2, or gestational diabetes when utilizing different diabetes monitoring systems (DMS). Future endeavors in personalized medicine will necessitate more extensive research to assess the effectiveness, cost-effectiveness, and comparative efficacy of DMS, allowing for stratification into the most suitable subgroups of diabetic patients (Kamusheva et al., 2021). Table 4 lists some diabetes management systems that have obtained clinical approval. By customizing treatments to individual patient profiles, which include genetic, environmental, and lifestyle factors, healthcare providers can enhance therapeutic outcomes and reduce adverse effects (Kalra et al., 2022). This concept is illustrated in Figure 14. The incorporation of artificial intelligence and machine learning into the analysis of patient data can substantially improve the accuracy of personalized treatment strategies (Clinton and Cross, 2023).

**TABLE 4:** Summarized some diabetes management systems that have obtained clinical approval.

Official title	Conditions	Intervention/Treatment	ClinicalTrials.gov ID	Study completion
A Randomized Cross-over Trial Evaluating Automated Insulin Delivery Technologies on Hypoglycemia and Quality of Life in Elderly Adults With Type 1 Diabetes	T1DM	• Device: Tandemt: slim X2 with HCL or PLGS	NCT04016662	2024–01
Use of the Guardian™ Connect System With Smart Connected Devices	T1DM	• Device: Guardian™ Connect system, InPen™ Basal smart cap, smart insulin pens, and InPen™ Diabetes Management app	NCT04809285	2023–09
Individualized Planned Eating Patterns to Improve Glycemic Management in Adolescents With Type 1 Diabetes: A Pilot Clinical Trial	T1DM	• Behavioral: “MyPlan” -Individualized Planned Eating Pattern	NCT05147324	2023–04
Randomized Controlled Trial To Assess the Benefits of Dexcom Continuous Glucose Monitoring With Glucose Telemetry System for the Management of Diabetes in Long-term Care Setting: The CGM-GTS in Long-term Care	T2DM	• Device: Dexcom G6 CGM with GTS • Diagnostic Test: POC Blood Glucose Test + Blinded CGM	NCT04818242	2022–10
ABC [Afrezza With Basal Combination]: A Phase 4 Study of Mealtime Control With Afrezza in Adult Subjects With Type 1 Diabetes Mellitus in Combination With an Automated Insulin Pump or Insulin Degludec	T1DM	• Biological: Afrezza (insulin human) Inhalation Powder • Biological: insulin degludec • Device: Continuous Subcutaneous Insulin Infusion (CSII) pump with Automatic Insulin Delivery (AID)	NCT05243628	2022–10
Hybrid Closed Loop Therapy and Verapamil for Beta Cell Preservation in New Onset Type 1 Diabetes (CLVer)	T1DM	• Device: HCL • Drug: verapamil 120 mg tablet • Device: non-HCL • Drug: placebo	NCT04233034	2022–09
Automated Insulin Delivery for INpatients With DysGlycemia (AIDING) Feasibility Study	DM	• Device: The Omnipod 5/Horizon HCL system	NCT04714216	2022–08
Feasibility of Outpatient Automated Blood Glucose Control With the iLet Bionic Pancreas for Treatment of Cystic Fibrosis Related Diabetes	Cystic Fibrosis-related Diabetes	• Device: Bionic Pancreas • Other: Usual Care	NCT03258853	2022–06
Demonstration Study of the Interest of the MEDTRUM A7+ TouchCare Insulin Patch Pump Versus INSULET Omnipod^®^ Patch Pump	DM	• Device: Medtrum A7+ insulin Pump • Biological: Lab A1C	NCT04223973	2021–06
QBSAfe: A Novel Approach to Diabetes Management Focused on Quality of Life, Burden of Treatment, Social Integration and Avoidance of Future Events	DM	• Other: QBSAfe Toolkit	NCT04514523	2020–09
Assessment of a Novel Sensing Catheter During Automated Insulin Delivery in Patients with Type 1 Diabetes	T1DM	• Device: Artificial Pancreas Control system (APC) • Device: Pacific Diabetes Technologies CGM Insulin Infusion system	NCT03528174	2018–07
ACCU-CHEK Connect Personal Diabetes Management Study (PDM)	T1DM	• Device: ACCU-CHEK	NCT02600845	2017–02
A Multicenter Study of Outpatient Automated Blood Glucose Control With a Bihormonal Bionic Pancreas	T1DM	• Device: Bionic Pancreas • Device: Insulin pump with or without CGM	NCT02092220	2016–12
The Mobile Insulin Titration Intervention (MITI) Study: Innovative Chronic Disease Management of Diabetes	DM	• Other: Mobile Insulin Titration Intervention	NCT01879579	2015–06
Diabetes Remote Care Management System	DM	• Device: DRMS	NCT01354015	2014–09
Sensor and Software Use for Improved Glucose Control in MDI Managed Diabetes	DM	• Device: FreeStyle Navigator • Device: Standard SMBG	NCT01713348	2013–07

Nevertheless, challenges still exist in terms of the accessibility of personalized therapies and the need for solid clinical evidence to support their efficacy across diverse populations (Varela-Moreno et al., 2021). Future research must concentrate on developing scalable models for personalized interventions that can be effectively implemented in clinical practice, ensuring that all patients can benefit from these advancements (Chen et al., 2024c; Huckvale et al., 2019; Lydiard and Nemeroff, 2019).

### 5.3 Importance of multidisciplinary collaboration

Multidisciplinary collaboration is essential for tackling the intricate challenges associated with the development and execution of biomaterials and personalized treatments. By uniting expertise from various fields, including engineering, biology, medicine, and data science, researchers can foster innovation and accelerate the application of scientific breakthroughs in clinical settings. Effective collaboration not only enhances research quality but also addresses the complex dimensions of health issues, leading to more holistic solutions (Errecaborde et al., 2019). For instance, collaborative initiatives in bioimage analysis have demonstrated the potential to enhance diagnostic precision and treatment planning (Schlaeppi et al., 2022). Furthermore, establishing standards for interprofessional collaboration can improve communication and cooperation among healthcare providers, ultimately leading to better patient outcomes (Bowman et al., 2021). As the healthcare landscape evolves, nurturing a culture of collaboration will be critical in overcoming obstacles and advancing the disciplines of biomaterials and personalized medicine.

## 6 Conclusion

The prevalence of diabetes, a prevalent metabolic disorder, is escalating globally. Conventional treatment modalities, such as pharmacological interventions and lifestyle modifications, often fall short of achieving optimal glycemic control due to issues like poor patient adherence and complex treatment protocols. There is an urgent need for innovative approaches.

The integration of multidisciplinary strategies will be vital for advancing biomedical research in the future. By merging perspectives from materials science, biomedical engineering, and clinical medicine, researchers can devise innovative solutions to tackle the multifaceted challenges posed by diabetes. Biomaterials encounter hurdles such as immune rejection, biocompatibility, and high costs in diabetes management applications. It is imperative to synthesize these findings through systematic reviews and meta-analyses, which can elucidate which materials and delivery systems are most likely to yield favorable outcomes for patients. This collaborative effort can facilitate the design of biomaterials that not only enhance insulin delivery and foster tissue regeneration but also prioritize patient safety and comfort.

In the future, it is necessary to strengthen research on the safety and effectiveness of biomaterials and establish standardized evaluation protocols; promote personalized treatment and formulate precise treatment plans according to individual differences of patients; strengthen multidisciplinary cooperation and promote the transformation of biomaterials from laboratory to clinic to improve the treatment effect and quality of life of diabetic patients.
